# Iron oxide decorated nitrogen doped carbon derived from iron MOFs and polyaniline as binder free electrode for symmetric supercapacitors

**DOI:** 10.1038/s41598-026-39173-4

**Published:** 2026-03-09

**Authors:** Aya A. El-Ashry, Dalia M. El-Gendy, Mina Shawky Adly, Ehab N. El Sawy, Sohier A. El-Hakam

**Affiliations:** 1 Chemistry Department, Faculty of Science, Mansoura University, 35516 Mansoura, Egypt; 2 Department of Chemistry, School of Sciences and Engineering, The American University in Cairo, New Cairo, 11835 Egypt; 3 Physical Chemistry Department, National Research Centre, Dokki, Giza, Egypt

**Keywords:** Nitrogen doping, MIL-101 (Fe), Fe_3_O_4_, PANI, Asymmetric supercapacitor, Chemistry, Energy science and technology, Materials science, Nanoscience and technology

## Abstract

Electrochemical supercapacitors are crucial energy storage technologies for the evolution of electric cars and the regulation/utilization of intermittent renewable resources. In his work, Fe_3_O_4_/nitrogen-doped carbon (FNC) was fabricated via an efficient, facile solvothermal synthesis of the iron-based metal-organic framework (MIL-101(Fe) and NH_2_-MIL-101(Fe)), followed by pyrolysis under a N_2_ atmosphere. The prepared FNC was mixed at different ratios with pyrolyzed polyaniline (P-PANI) to enhance the conductivity, hydrophilicity, and electrochemical performance of the fabricated electrode materials. The morphology and crystallinity of the synthesized FNC@P-PANI composites were evaluated by SEM and XRD, revealing a nanoscale architecture with good crystallinity. The electrochemical behavior of FNC, P-PANI, and FNC@P-PANI materials was studied using cyclic voltammetry (CV), galvanostatic charge-discharge (GCD), and electrochemical impedance spectroscopy (EIS) in 1.0 M Li_2_SO_4_ as a neutral electrolyte. Compared to plain FNC and P-PANI, the 20FNC@P-PANI composite showed an enhanced specific capacitance of 633.9 F/g at 1 A/g. Furthermore, the assembled device exhibited outstanding stability of 100.1% after 1,000 cycles and 95.86% after 10,000 cycles at 10 A/g. The 20FNC@P-PANI composite’s high surface area, stability, and rapid ion and electron transport facilitated its high electrochemical efficacy. Moreover, the symmetric supercapacitor device delivered superior energy and power densities of 47.52 Wh/kg and 789.99 W/kg, respectively, at 1 A/g. These favorable results suggest that it is possible to produce innovative, ecologically friendly, and commercial electrode materials based on Fe_3_O_4_ and carbon-doped nitrogen.

**Supplementary Information:**

The online version contains supplementary material available at 10.1038/s41598-026-39173-4.

## Introduction

Human energy consumption, primarily from fossil fuels, contributes to pollution, global warming, and elevated CO_2_ emissions. There’s growing interest in renewable energy sources such as wind, solar, and tidal energy, alongside rechargeable electrochemical storage systems, as traditional energy sources are depleted. Energy storage solutions such as lithium-ion batteries (LIBs), supercapacitors (SCs), and electrolyzers are vital for efficiently storing and supplying energy. SCs are gaining attention for their long cycle life, high power density, and fast charging/discharging^1^. They combine the high power of capacitors with the energy of batteries, offering longer cycle life and higher energy density than Li-ion batteries, thereby addressing energy challenges.

SCs are categorized as electrical double-layer capacitors (EDLCs), pseudocapacitors (PCs), and hybrids. Carbon nanomaterials, such as graphene and nanotubes, are used in EDLCs for their large surface areas, whereas metal oxides and conductive polymers are used in PCs, with surface area, porosity, conductivity, and pore structure being key parameters that affect their performance^2^.

Metal-organic frameworks (MOFs) possess a high surface area, tunable porosity, and controllable structures. These attributes facilitate their application in energy storage (e.g., supercapacitors and lithium-ion batteries), gas separation, catalysis, and sensing. Nonetheless, some MOFs demonstrate limited stability, low electrical conductivity, and minimal capacitance. To mitigate these issues, carbon materials derived from pyrolyzed MOFs have shown excellent performance in both symmetric and asymmetric supercapacitors. MOFs serve as templates for synthesizing metal/metal oxide and porous carbon materials with high surface areas and well-dispersed pore structures, which are crucial for charge storage. For instance, Fe-MIL-101 is utilized to produce iron-based catalysts for energy storage applications. Thermal treatment in a nitrogen atmosphere results in the formation of metallic iron, iron oxides, and carbides, though FeN_x_ is not formed due to the high iron and low nitrogen content. The development of mesoporous MIL-101(Fe) carbons with external nitrogen functionalization presents a promising avenue for supercapacitor applications. Nitrogen-rich sites, introduced via amine-functionalized MOFs, improve mass transport and enhance supercapacitor performance in neutral and alkaline electrolytes. Additionally, carbon materials contribute through edge defects, efficient electron-transport pathways, and catalytically active oxygen functionalities. Incorporating heteroatoms, such as nitrogen, sulfur, and phosphorus, further enhances the performance of fabricated supercapacitors.

Conducting polymers, known for their excellent electrical and optical properties and ease of fabrication, have gained widespread attention in various fields. Conductive polymers (CPs) have recently attracted much attention for different applications, including energy storage and conversion, supercapacitors, catalysis, and biosensors, due to their large surface area and electrical conductivity, as well as their excellent chemical and physical stability^3,4^. These polymers possess conjugated structures that enable electrical conductivity through delocalized π electrons. However, the inherent instability of these polymers, due to bond alterations that introduce energy gaps, limits their practical use^5,6^. Polyaniline (PANI) is notable for its stability, versatility, affordability, easy synthesis, moderate electrochemical activity, multiple oxidation states, and tunable properties^4,7,8^. It is a common electrode material that can be readily proton-doped and exhibits high capacity^9^. PANI exists in various oxidation states, such as pernigraniline, emeraldine base, and leucoemeraldine, with emeraldine base being the most conductive and stable. Optimizing its concentration improves supercapacitor performance. Synthesized via chemical or electrochemical oxidation, PANI yields morphologies such as nanotubes, nanorods, nanospheres, and nanofibers^10,11^. Pyrolysis of PANI produces nitrogen-doped carbon materials with high surface area and porosity, which are attractive for supercapacitors that depend on surface area, uniform dispersion, and high conductivity^12^.

This study synthesized Fe_3_O_4_/nitrogen-doped carbon (FNC) *via* a simple hydrothermal method using NH_2_-MIL-101(Fe) MOFs, followed by pyrolysis under N_2_ gas. The resulting FNC nanoparticles were then combined with varying ratios of pyrolyzed polyaniline (P-PANI) to improve the electrode materials’ conductivity, hydrophilicity, and electrochemical performance. The electrochemical properties of the fabricated electrodes were evaluated using cyclic voltammetry (CV), galvanostatic charge-discharge (GCD), and electrochemical impedance spectroscopy (EIS). The optimized electrode exhibited excellent electrochemical performance, achieving a specific capacitance of 633.9 F/g at a current density of 1 A/g in a three-electrode setup using 1.0 M Li_2_SO_4_ as the electrolyte.

## Experimental

### Materials

All chemicals employed in the fabrication of electrode materials were used without further purification. Ferric chloride hexahydrate (FeCl_3_ 6H_2_O), terephthalic acid (Benzene-1,4-dicarboxylic acid, BDC), and 2-aminoterephthalic acid (NH_2_-BDC) were purchased from Sigma-Aldrich. Aniline, hydrochloric acid (HCl), ammonium persulfate (APS), and lithium sulfate (Li_2_SO_4_) were obtained from Loba Chemie. Deionized water with a resistivity > 18.3 MΩ cm was used to prepare the electrolyte solution.

### Preparation of MIL-101(Fe) and NH_2_-MIL-101(Fe)

MIL-101(Fe) is synthesized following a previously reported method with slight modifications^13^. Typically, 1.35 g (3.38 mmol) of FeCl_3_. 6H_2_O was dissolved in 30 mL of DMF under stirring, followed by the addition of 0.41 g (2.47 mmol) terephthalic acid. The resultant mixture was introduced into a 100 mL Teflon-lined autoclave, then transferred to an oven at 110 °C, and maintained at that temperature for 20 h. An orange powder was obtained and separated by centrifugation at 4,000 rpm. The obtained powder was washed using hot DMF, then ethanol, each for 3 times, and dried at 80 °C overnight. The NH_2_-MIL-101(Fe) was synthesized following a similar procedure to that of MIL-101(Fe), but terephthalic acid was replaced by 0.434 g (2.4 mmol) of 2-aminoterephthalic acid.

### Preparation of PANI

The polyaniline (PANI) micro/nanostructures were synthesized using a simple chemical oxidative polymerization technique, as follows: 3.17 mL of aniline mixed with 60 mL HCl (1.0 M) for 30 min in an ice bath, then 0.913 g of APS was added into the solution and allowed to further stir for four hours^14^. The solution turned into a dark green suspension, indicating the complete polymerization of aniline. The suspension was then collected by centrifugation and washed several times with ethanol and water. Finally, the obtained precipitate was dried overnight at 80 °C and stored for further use.

### Preparation of FNC@P-PANI nanocomposites

To prepare the NH_2_-MIL-101(Fe)@PANI composite, the NH_2_-MIL-101(Fe) and PANI were mixed and hydrothermally treated to be chemically bonded. Firstly, the calculated amounts of PANI and NH_2_-MIL-101(Fe) were suspended in two separate beakers containing 30 mL of DI water for 30 min using an ultrasonication water bath. Subsequently, the two suspensions were mixed and transferred to a stainless-steel autoclave, which was then heated to 170 °C and held for 24 h. After cooling, the resulting powder was segregated by centrifugation, washed several times with water, and dried at 70 °C. The percentage of NH_2_-MIL-101(Fe) used to prepare the NH_2_-MIL-101(Fe)@PANI composite was 10, 20, and 30 wt% of the total mass. All prepared composites were thermally pyrolyzed in a tube furnace under N_2_ at 500 °C for 1 h, with a ramp rate of 5 °C/min. The MIL-101(Fe), NH_2_-MIL-101(Fe), PANI, 10 wt% NH_2_-MIL-101 (Fe)@PANI, 20 wt% NH_2_-MIL-101(Fe)@PANI, and 30 wt% NH_2_-MIL-101(Fe)@PANI pyrolyzed samples were labeled as FC, FNC, P-PANI, 10FNC@P-PANI, 20FNC@P-PANI, and 30FNC@P-PANI nanocomposites, respectively.

### Characterization

Several analytical methods were employed to confirm the successful fabrication of the composites and to monitor the physical and chemical changes that occurred after pyrolysis. A JASCO spectrometer (FTIR-6300 type A) spectrophotometer with a total reflectance attenuated by a diamond (FT-DATR), in wavenumber spanning from 4000 to 500 cm^− 1^, was used to examine the functional groups in the fabricated nanocomposites before and after pyrolysis. Additionally, the Bruker D8 Advance X-ray diffractometer (λ = 1.54178 Å, step size of 0.02, scan step of 0.80 s, and 2θ of 5°-70°) was used to gather the crystal configurations of the generated materials. X-ray photoelectron spectroscopy (XPS) measurements were performed using a Thermo Scientific ESCALAB 250Xi equipped with a monochromatic Al K_α_ source to investigate the surface chemical composition and electronic states of the materials. Raman data measured using a confocal Raman microscope (WITec Alpha 300RA), 532 nm excitation laser source, and 5 mW laser power. Thermal gravimetric analysis (TGA) was performed on the powder samples over the temperature range from room temperature to 600 °C under N_2_ at a heating rate of 10 °C/min using a Shimadzu TGA-50/50H.

### Electrochemical measurements

All electrochemical measurements were performed using a BioLogic SP-150e Potentiostat/Galvanostat, accompanied by EC-Lab V11.43 software (https://www.biologic.net/softwares/ec-lab-software/) for data acquisition. All experiments were repeated three times, and the representative data were used for comparison. EC-Lab V11.43 software was used to fit electrochemical impedance spectroscopy (EIS) data. A typical three-electrode setup was used, featuring a gold wire and an Hg/HgSO_4_/Sat. K_2_SO_4_ (Mercury sulfate electrode, MSE (Eº = 0.64 versus SHE) as the auxiliary and the reference electrodes, respectively, and 1.0 M Li_2_SO_4_ solution as an electrolyte. The working electrode consisted of a graphite sheet (3 mm thickness) with an active area of 1 $\:\times\:$ 1 cm^2^, which was coated with an ink of the desired material using an airbrush. The ink of the active material was prepared by dispersing 5 mg of the active material in 500 µL DI water using sonication for 30 min. The fabricated electrodes were dried under vacuum at 60 °C for 1 h at a material loading of approximately 0.5 mg/cm^2^. The working electrodes were activated using 50 CV cycles at a scan rate of 50 mV/s within the potential range under study.

EIS measurements were acquired at an amplitude of 5 mV and a frequency range of 0.01 Hz to 100 kHz under open circuit potential (OCP). CV measurements were performed at scan rates from 5 to 100 mV/s over the potential ranges − 0.9 to 0 V at the negative electrode and 0 to 0.7 V at the positive electrode. The galvanostatic charge-discharge (GCD) studies were performed after the CV measurements within similar potential windows and at different current densities of 1, 3, 5, and 10 A/g. The specific capacitance (C_s_) values, in F/g, were evaluated from CV and GCD curves according to Eqs. 1 and 2, respectively^15–17^.

1 $$\:{\mathrm{C}}_{\mathrm{s}}=\frac{\int\:\mathrm{I}\mathrm{d}\mathrm{V}}{{\upnu\:}\mathrm{m}\:\:\varDelta\:\mathrm{V}}$$

Where **I** (A) is current, $\:\nu\:$ (V/s) is the scan rate, **m** (g) is the mass of the active material, and **∆V** (V) is the potential window of the CV curves^18,19^.

2 $$\:{\mathrm{C}}_{\mathrm{s}}=\frac{\mathrm{I}\:\varDelta\:\mathrm{t}}{\mathrm{m}\:\varDelta\:\mathrm{V}}$$

Where **I** (A) is the discharge current, **∆t** (s) is the discharge time, **m** (g) is the mass of active material, and **∆V** (V) is the discharge potential range.

In assembling the symmetric supercapacitor (SSC), 20FNC@P-PANI was used as both the positive and negative electrodes, with filter paper as the separator. The performance of the SSC device was evaluated using CV at various scan rates of 5-100 mV/s and a potential window of 1.6 V, GCD at different current densities of 1, 3, 5, and 10 A/g at a potential window similar to that used for the CV measurements, and EIS measurements at OCP, using an amplitude of 5 mV and a frequency of 0.01–10^5^ Hz.

The energy density (ED) in Wh/kg and the power density (PD) in W/kg were calculated using Eqs. 3 and 4, respectively^20,21^.

3 $$\:\mathrm{E}\mathrm{D}=\frac{1}{2}{\mathrm{C}}_{\mathrm{s}}{\left(\varDelta\:\mathrm{V}\right)}^{2}=\frac{\mathrm{I}\varDelta\:\mathrm{V}\mathrm{t}}{2\mathrm{m}}$$

4 $$\:\mathrm{P}\mathrm{D}=\frac{\mathrm{E}}{\mathrm{t}}=\frac{\mathrm{I}\varDelta\:\mathrm{V}}{\mathrm{m}}$$

Where **C**_**s**_ (F/g) is the specific capacitance, **V** (V) is the potential window, and **t** (s) is the discharge time.

## Results and discussion

### Characterization

**Fig. 1:**
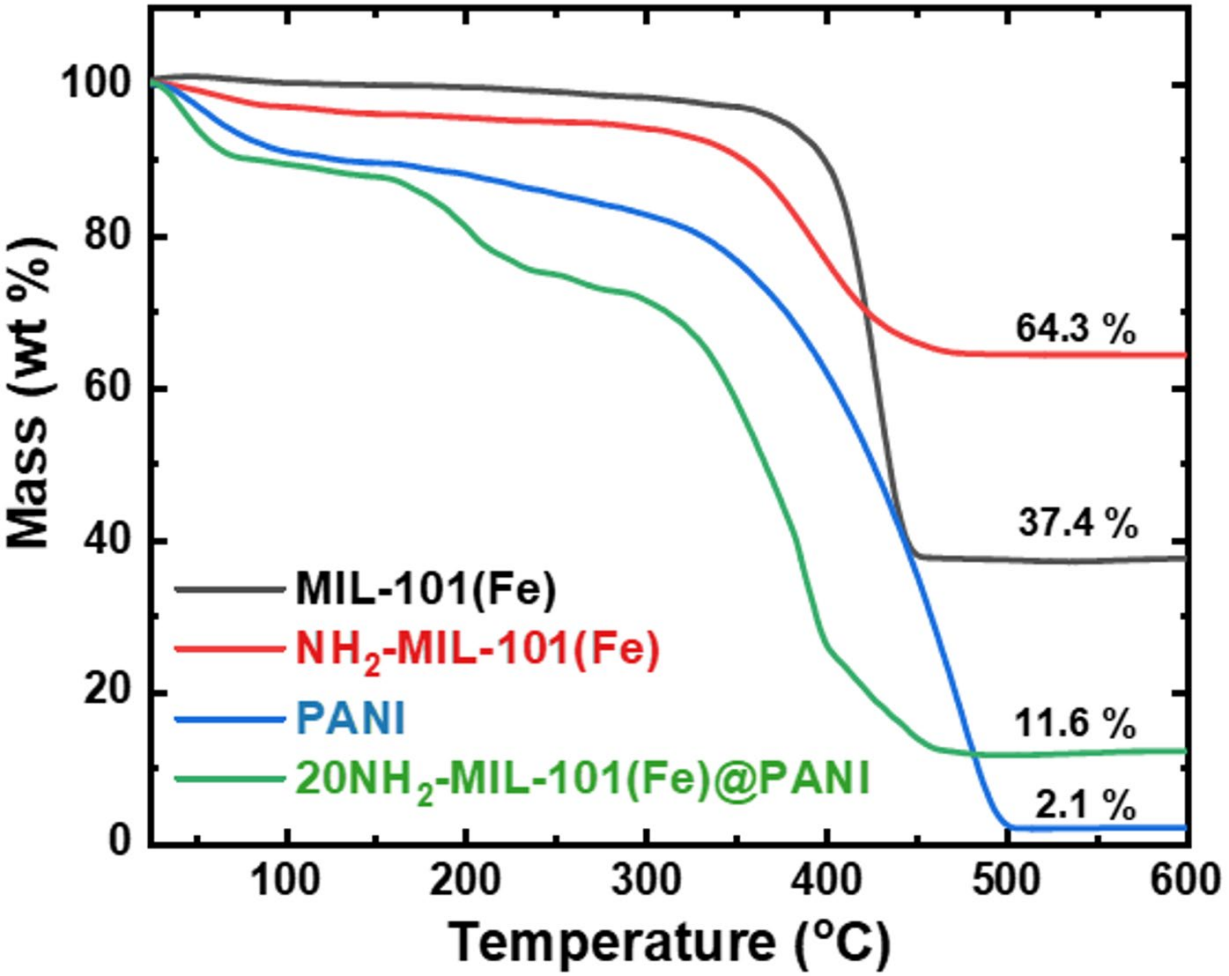
Thermogravimetric analysis (TGA) of the as-prepared MIL-101(Fe), NH_2_-MIL-101(Fe), PANI, and 20NH_2_-MIL-101(Fe)@PANI under nitrogen gas, using a temperature ramp of 10 °C/min.

Figure 1 displays the thermogravimetric analysis of the bare Fe-MOFs (MIL-101(Fe) and NH_2_-MIL-101(Fe)), which shows two-step mass losses at < 100 °C and 340–450 °C. The first loss can correspond to the removal of adsorbed water and/or to the volatilization of guest molecules within the framework structure^22^. The second loss is attributed to the pyrolysis of the organic ligands, the removal of solvent molecules from the sample’s surface, and the disintegration of the bond between the metal core within the pores and the functional groups^23^. Both Fe-MOFs exhibit high thermal stability, with NH_2_-MIL-101(Fe) showing greater stability due to the incorporation of the amino group into the organic linker^24^. For PANI, the thermograph also shows two-step mass losses: the first (< 100 °C) is attributed to the volatilization of adsorbed water molecules, and the second (340–500 °C) to the decomposition of the polymer chain^61^. In the case of the 20PANI@NH_2_-MIL-101(Fe) composite, a third step is observed at 160–280 °C and a rapid mass loss at 300- to 400 °C, indicating better stability of PANI and NH_2_-MIL-101(Fe) in comparison to their composite^25^. These data suggest that the pyrolysis of the Fe-MOFs, PANI, and their composites at 500 °C was enough to convert them to stable Fe_3_O_4_-decorated nitrogen-doped carbon.

**Fig. 2:**
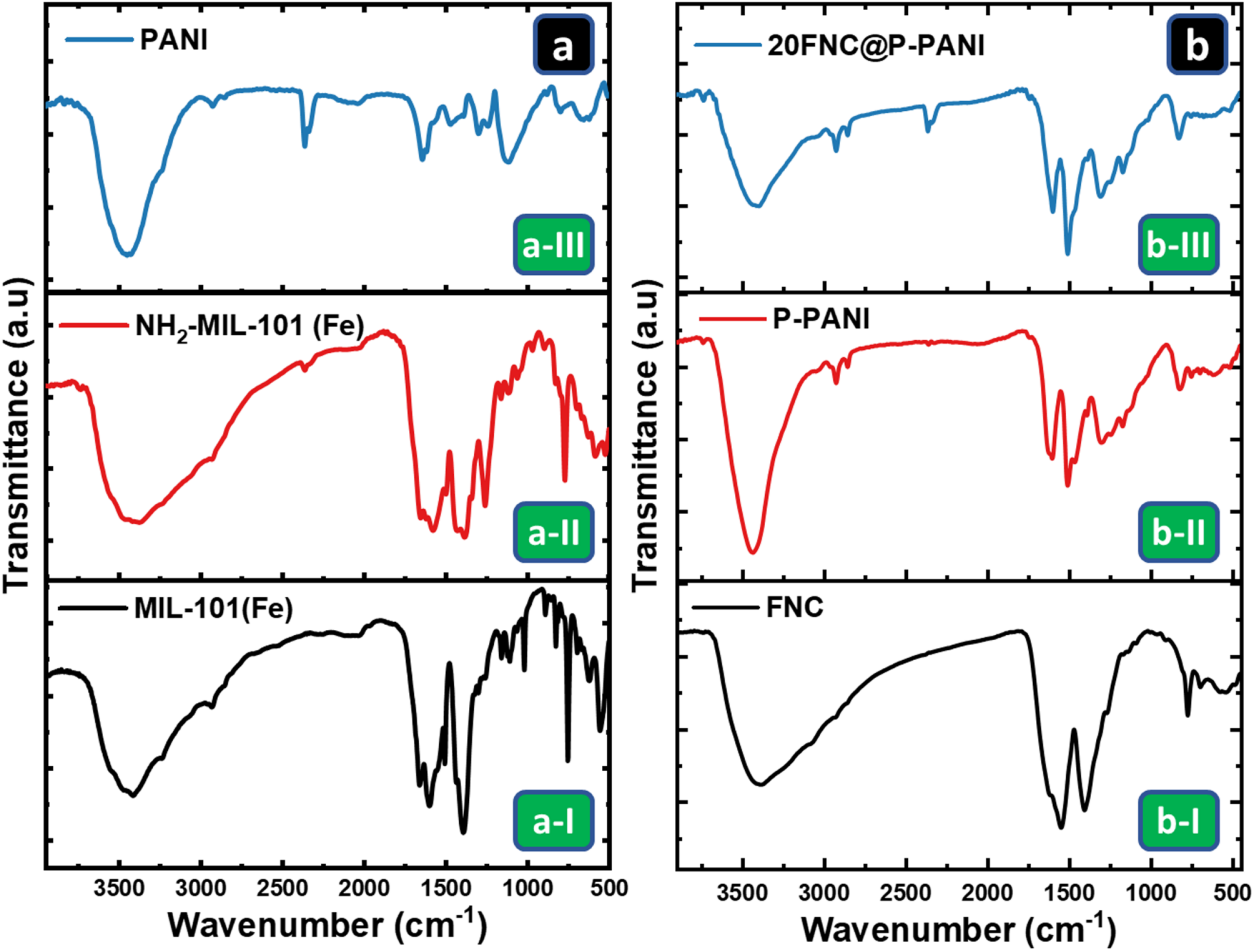
FTIR spectra of the synthesized material before (**a**) and after (**b**) pyrolysis are as follows: (a-I) MIL-10(Fe), (a-II) NH_2_-MIL-101(Fe), (a-III) PANI, (b-I) FNC, (b-II) P-PANI, and (b-III) 20FNC@P-PANI.

The functional groups and chemical bonds of the generated composites were examined using FT-IR spectroscopy. The FTIR spectra of the fabricated Fe-MOFs (MIL-101(Fe) and NH_2_-MIL-101(Fe)) and PANI are depicted in Fig. 2a-I and a-II, and 2a-III, respectively. In Fig. 2a-I and a-II, the band allocated at 554–585 cm^− 1^ is assigned to the Fe$\:-$O bond, and the band at 747–771 cm^− 1^ is assigned to the C$\:-$H aromatic ring stretching vibrations in the terephthalic linker^26^. Furthermore, the bands at 1578–1608 and 1385–1393 cm^− 1^ are related to the asymmetric and symmetric vibrations of the carboxyl group, respectively, and the band at 1429 –1426 cm^− 1^ is related to the aromatic carbon C$\:=$C vibrational mode. The band at around 1652–1665 cm^−1^ is associated with the vibration of carboxylate groups, and its relatively high intensity suggests a significant number of free carboxylic groups^27^. A broad band at 3403 cm^− 1^ is observed due to the O$\:-$H and N$\:-$H stretching overlap^26,28^. The presence of the amino group in the NH_2_-MIL-101(Fe) sample is confirmed by the two small bands assigned at 3455 and 3380 cm^− 1^ related to the symmetric and asymmetric stretching vibrations of the amino group, and the band at 1265 cm^− 1^ due to the stretching vibration of the aromatic carbon C–N bonding^29^.

For the PANI spectrum in Fig. 2a-III, two bands at 1572 and 1469 cm^− 1^ are ascribed to the C$\:=$C stretching vibration modes of quinoid and benzenoid rings in the polymer chains, respectively. The band allocated at 1300 cm^−1^ is attributed to the C$\:-$N stretching vibration, and those at 1117 and 795 cm^− 1^ are attributed to the in-plane and out-of-plane bending vibrations of C$\:-$H, respectively^30–33^.

In Fig. 2b-I, the FNC nanocomposite shows different bands than those observed in the NH_2_-MIL-101(Fe) spectrum (Fig. 2a-II). The two bands at 554–585 cm^− 1^ observed in the case of NH_2_-MIL-101(Fe), related to the Fe–O group, were replaced by a new peak at 550 cm^− 1^ upon the FNC nanocomposite formation. The bands located at 1652–1665 cm^− 1^ in NH_2_-MIL-101(Fe) were replaced with a band at 1555 cm^− 1^ in FNC due to the symmetric and asymmetric bending vibration of the C$\:=$O. The strong band below 700 cm^−1^ is assigned to Fe$\:-$O stretching mode^34^. The differences in the feature bands at 1545 and 1150 cm^− 1^ were ascribed to CO stretching vibrations resulting from the pyrolysis of NH_2_-MIL-101(Fe).

The spectrum of the P-PANI (Fig. 2b-II), generated from the pyrolysis of PANI, suggests that it is partially converted into a new nitrogen-doped carbon structure. The bands at 3400 and 2925 cm^− 1^ are assigned to the $\:-$OH and/or $\:-$NH stretching and C$\:-$H stretching, respectively^35–37^. The band at 1614 cm^− 1^ corresponds to the C$\:=$C stretching vibration of the quinoid ring and the bands related to the C$\:-$H in-plane bending are allocated at 1493 and 1396 cm^−1^. The C-N and the N$\:=$N stretching bands at 1310 cm^− 1^ and 1162 cm^−1^, respectively, are characteristic bands of the PANI base^35,36^.

In the case of 20FNC@P-PANI, the FTIR spectrum in Fig. 2b-III shows the characteristic band at 3430 cm^− 1^ associated with O–H stretching vibrations, indicating the presence of adsorbed water molecules. Interestingly, most typical bands associated with P-PANI and Fe-MOFs (FNC) were observed in the produced composites with lower PANI concentrations^38^. The bands at $\:\sim$1570 and 1150.9 cm^−1^ were ascribed to C$\:=$N and C–N stretching vibrations, respectively. These results indicate that the addition of PANI could simultaneously enable nitrogen atoms to be inserted into the final sample during pyrolysis^39^. The band at 530 cm^− 1^ is attributed to Fe$\:-$O stretching vibration.

**Fig. 3:**
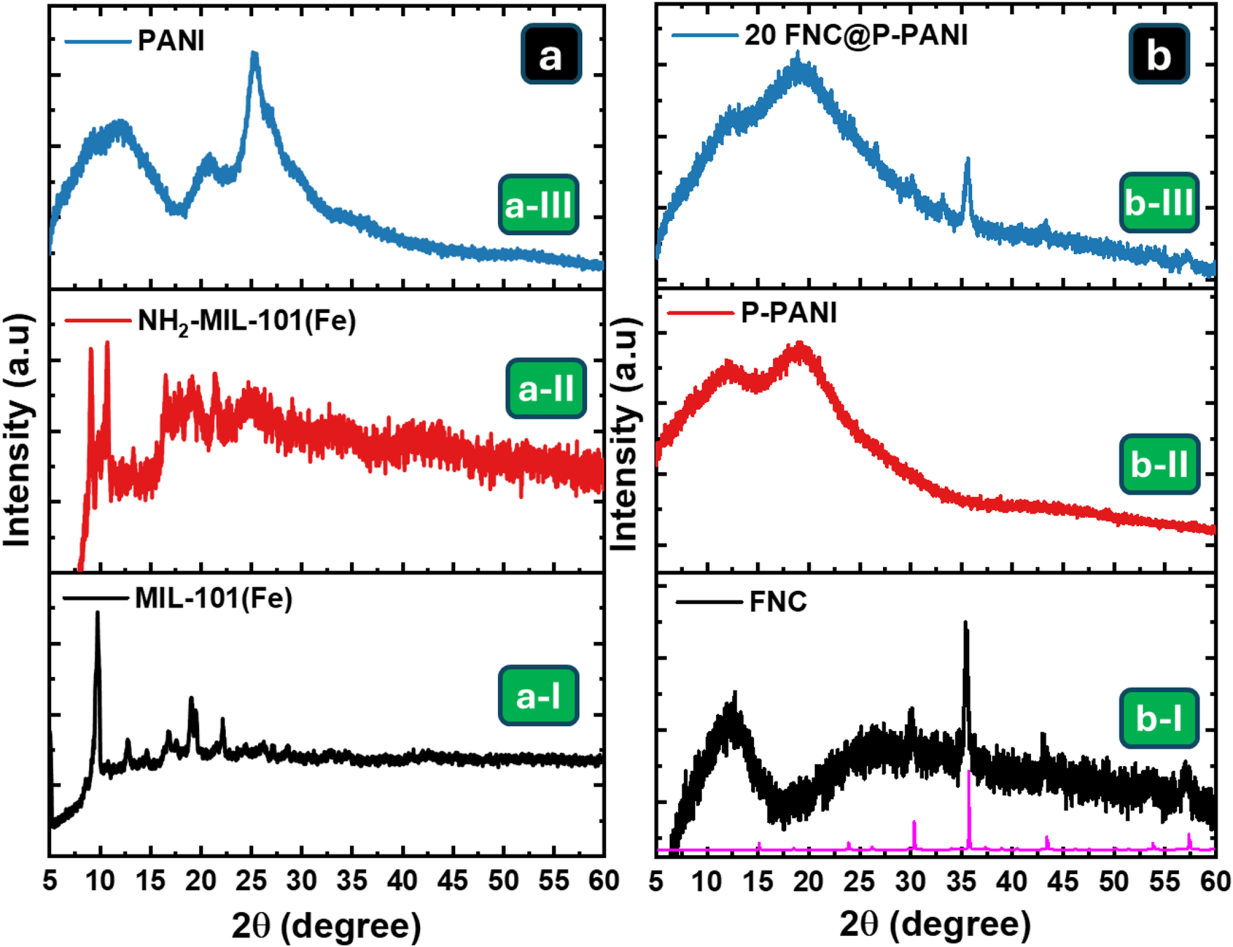
XRD patterns of the synthesized material before (**a**) and after (**b**) pyrolysis are as follows: (a-I) MIL-101(Fe), (a-II) NH_2_-MIL-101(Fe), (a-III) PANI, (b-I) FNC, (b-II) P-PANI, and (b-III) 20FNC@P-PANI.

Figure 3a-I shows the XRD pattern of the MIL-101(Fe), exhibiting well-defined diffraction peaks at 2θ of 9.8°, 12.7°, 16.7°, 19.1°, and 22.2°, which indicate the high crystallinity of the fabricated frameworks^40^. The NH_2_-MIL-101(Fe) pattern in Fig. 3a-II exhibits high-intensity diffraction peaks at 9.2º and 10.8º and low-intensity diffraction peaks at 13.3º and 21.4º, consistent with previous studies^41^. The XRD pattern of PANI shows three diffraction peaks at 2θ of 11.7, 20.7, and 25.5º, corresponding to the (322), (113), and (121) crystal planes, respectively^42^. These peaks may be related to scattering from polymer chains at interplanar spacing. The diffraction peak at 25.5º indicates that the PANI was synthesized with a reasonable degree of crystallinity^42^. The XRD pattern of the pyrolyzed Fe-MOFs (FNC) in Fig. 3b-I shows significant peaks related to Fe_3_O_4_ at 2θ of 30.1º, 35.6º, 43.4º, 53.6º and 57.2º, corresponding to Bragg reflections of (220), (311), (400), (422), and (511), respectively, reflecting the cubic inverse spinel structure of the Fe_3_O_4_ nanoparticles matched with standard JCPD (00-040-1139)^43^. The sharp characteristic peaks of the XRD patterns indicate the crystallization of Fe_3_O_4_ nanoparticles. The XRD pattern of P-PANI in Fig. 3b-II shows two distinct peaks at 12.6° and 19.0°, illustrating its amorphous structure (25). The XRD pattern of the 20FNC@P-PANI composite shows diffraction peaks similar to those of FNC and P-PANI with two extra minor peaks at 26.4° and 33.1° related to (012) and (014) crystal planes, reflecting the presence of a tiny portion of Fe_2_O_3_ that formed due to the interaction between the PANI and NH_2_-MIL-101(Fe), as illustrated in Fig. 3b-III.

**Fig. 4:**
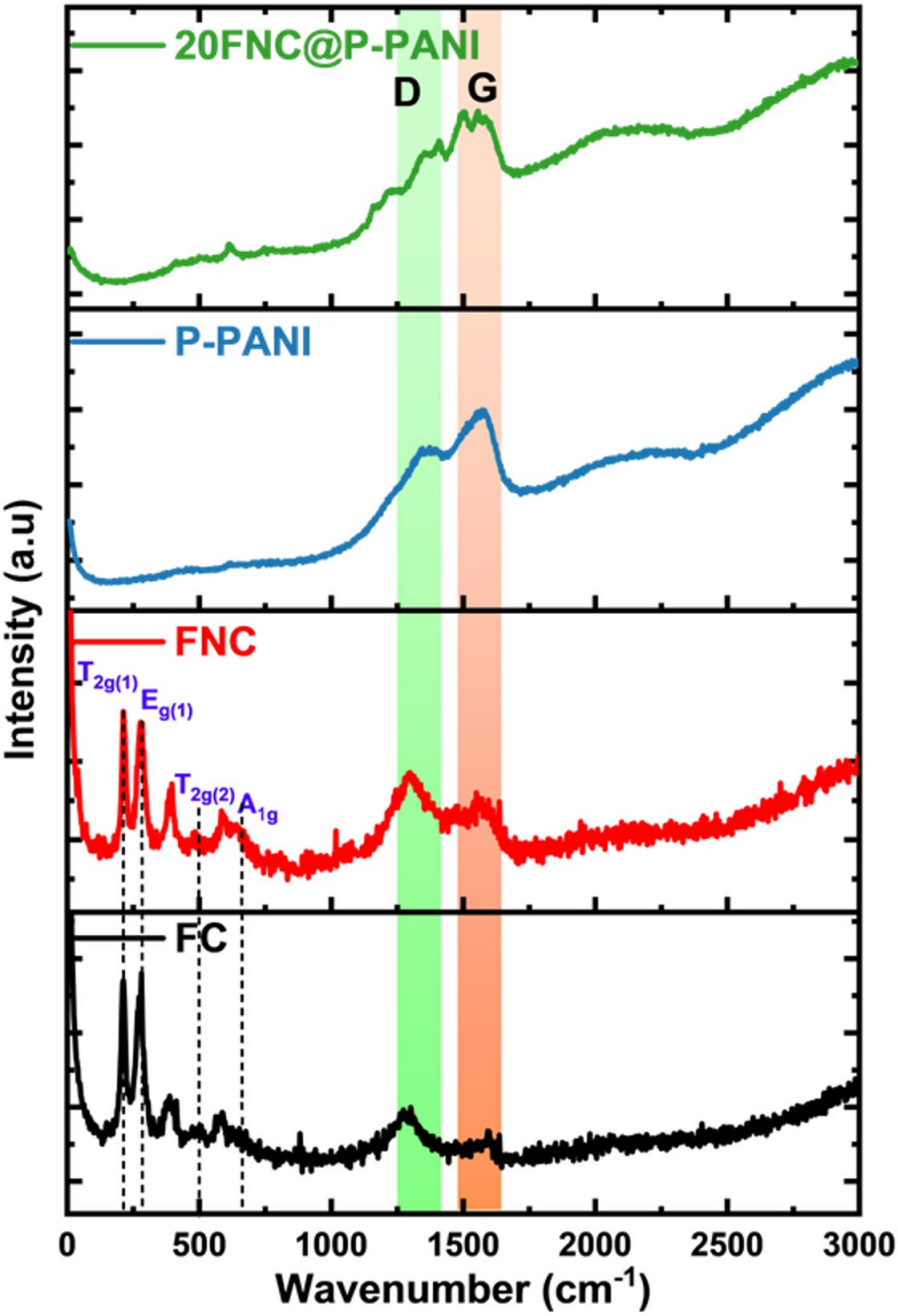
Raman spectroscopy of FC, FNC, P-PANI, and 20FNC@P-PANI.

The Raman spectroscopy of the fabricated electrode materials is shown in Fig. 4. Iron oxides are typically classified as materials that require careful handling during Raman spectroscopy because of the divalent transition metal (iron) in their structure, whether in the ferrous or ferric state^62^. The spectra showed peaks at 1295 and 1335 cm^− 1^, corresponding to the D band of Fe_3_O_4_-derived frameworks and 20FNC@P-PANI, respectively. Three peaks can be seen at approximately 397, 481, and 584 cm^− 1^, which correspond to the Fe-O bond vibration modes of FC and FNC composites. The oxidation process that took place during the Raman experiment is responsible for the peaks at 213 and 270 cm^− 1^^44^. Group theory predicts the following modes: Ag + Eg + 3T_2g_ are Raman-active^45^. The inverse degree of crystallinity of the materials will be closely correlated with the Raman spectrum. P-PANI is characterized by two peaks at 1346 and 1576 cm^− 1^, attributed to D and G bands. The D band indicates disordered structural flaws arising from carbonization. In contrast, the G band is assigned to the ordered arrangement of carbon atoms in a hexagonal two-dimensional structure due to C = C bond stretching. The D band in the carbon-carbon spectrum is associated with the double resonance phenomenon of disordered carbon structures. In contrast, the G band pertains to the in-plane vibrations of ordered graphite^46^. The integration ratio of the D and G bands (I_D_/I_G_=0.87) relative to P-PANI and 20FFNC@P-PANI demonstrated an increase in carbon defects due to structural damage.

**Fig. 5:**
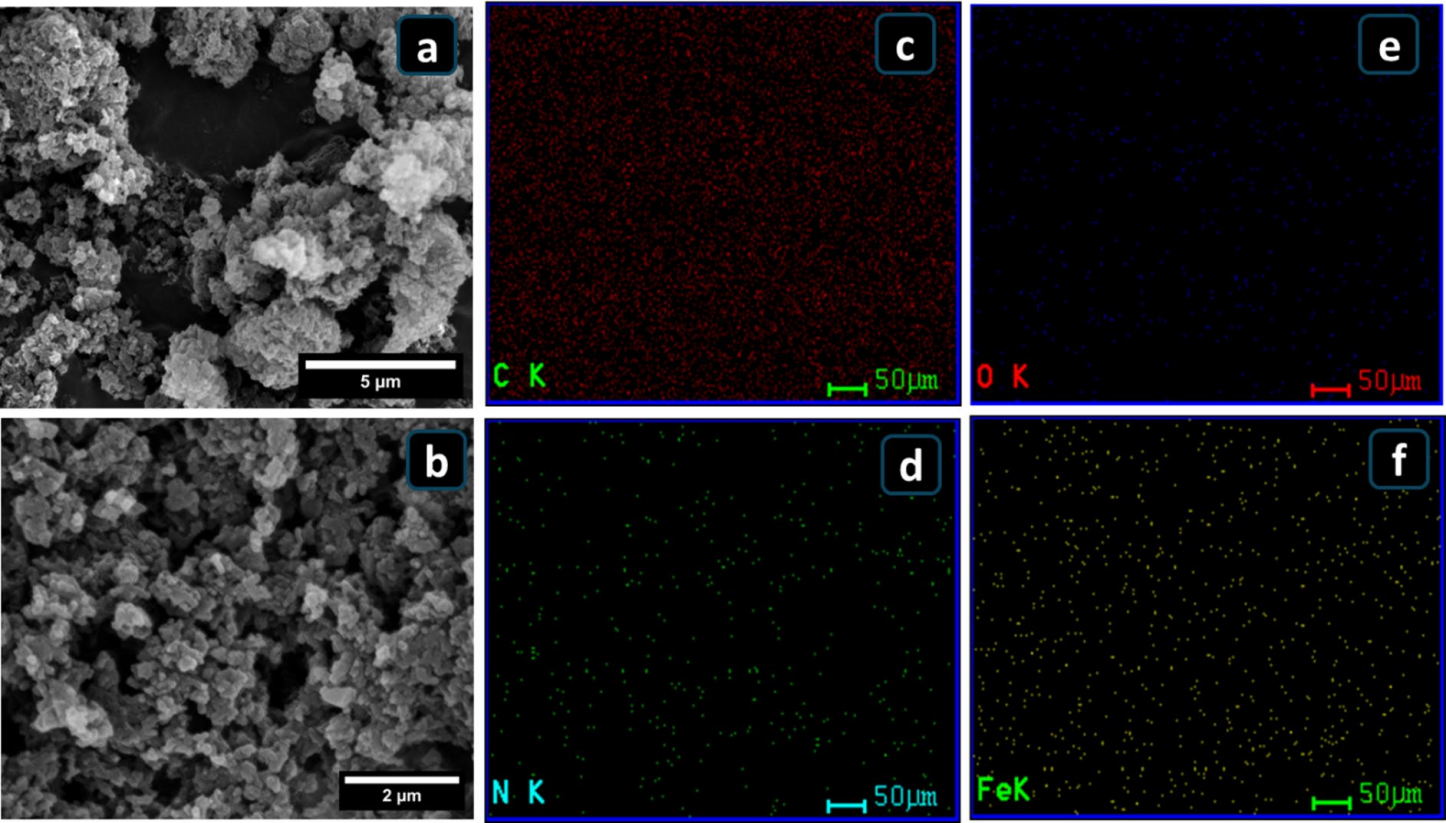
(**a** and **b**) SEM images, and (**c**-**f**) Energy dispersive X-ray spectroscopy (EDS) elemental maps (C, N, O, and Fe) of the 20FNC@P-PANI composite.

The morphology of the synthesized 20FNC@P-PANI nanocomposite is shown in Figs. 5a and b. The thermal treatment of the generated porous nanocomposites yields an acceptable surface area, thereby enhancing the diffusion of electrolyte ions. In addition, Fe_3_O_4_ nanoparticles were observed with slight aggregation, suggesting that good dispersion of the Fe_3_O_4_ nanoparticles was achieved on the surface and within the pyrolyzed PANI. EDX elemental mapping data are obtained and displayed to validate the composite structure. The elemental analysis results for iron and carbon are compatible with the raw SEM images, suggesting that the composite structure was successfully fabricated and distributed uniformly^47^. According to the EDS results in Figs. 5c-f, the elemental mapping images confirm that C, N, O, and Fe are uniformly distributed in the 20FNC@P-PANI composite, with atomic percentages of 80.9%, 5.6%, 11.5%, and 2%, respectively. This finding aligns with the EDS spot analysis presented in Figure S1 and summarized in Table S1.

**Fig. 6:**
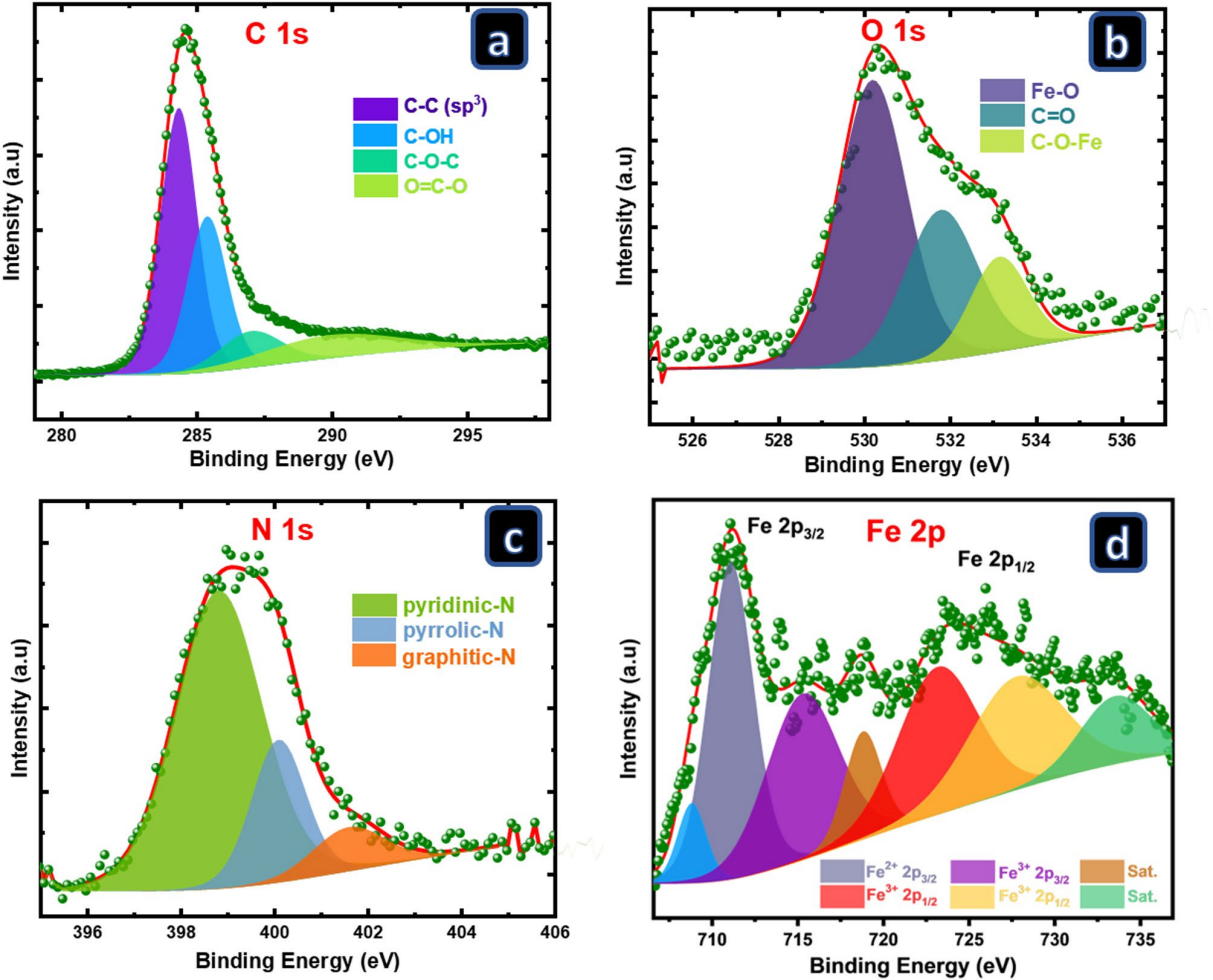
HR-XPS spectra of 20FNC@P-PANI showing the (**a**) C 1s, (**b**) O 1s, (**c**) N 1s, and (**d**) Fe 2p.

The chemical valence and composition of elements present on the surface of 20FNC@P-PANI were investigated *via* XPS analysis. The XPS spectrum for the fast survey in Figure S2 shows C 1s, O 1s, N 1s, and Fe 2p, confirming the successful preparation of the desired electrode (Fig. 6). In addition, the XPS spectrum shows several distinct energy peaks in the 1000–1100 eV region attributed to photoemission and Auger electron emission. The HR-XPS of C 1s is deconvoluted into four peaks allocated at 284.4 eV, 285.5 eV, 287.2 eV, and 290.2 eV, attributed to C$\:-$C, C$\:-$OH, C$\:-$O$\:-$C, and O$\:=$C$\:-$O, respectively^48,49^. The presence of the C$\:-$C (sp^2^) peak reflects the presence of graphitic structure and indicates the success of the pyrolysis process. The presence of peaks related to oxygenated functional groups (C$\:-$OH, C$\:-$O$\:-$C, and O$\:=$C$\:-$O) should result in a composite with enhanced hydrophilicity. The HR-XPS spectrum of O 1s splits into three peaks at 530.2, 531.8, and 533.1 eV related to Fe$\:-$O, C$\:=$O, and C$\:-$O$\:-$Fe, respectively^50^. The presence of a C$\:-$O$\:-$Fe peak indicates the homogeneous mixing and surface interaction of the Fe_3_O_4_ nanoparticles with the pyrolyzed PANI network.

The HR-XPS spectrum of the N 1s spectrum shows three prominent peaks characteristic of pyridinic-N (398.3 eV), pyrrolic-N (399.6 eV), and graphitic-N (400.9 eV), demonstrating that the electrocatalyst had been effectively doped with nitrogen atoms. Pyridinic-N is known to play a crucial role in forming Fe/N/C active sites with modified electronic structures. It has been demonstrated that pyridinic-N is advantageous for electronic conductivity because of the creation of a localized donor state and the higher state density of the Fermi surface^51^. The HR-XPS spectrum of Fe 2p is characterized by two prominent peaks for Fe 2p_3/2_ and Fe 2p_1/2_ at binding energies of 711.2 eV and 723.8 eV, respectively, which are deconvoluted into seven peaks. The HR-XPS of Fe 2p was found to be split into Fe 2p_3/2_ and Fe 2p_1/2_ peaks at 711.1 and 723.4 eV, corresponding to Fe^2+^; similarly, Fe 2p_3/2_ and Fe 2p_1/2_ peaks of Fe^3+^ were found at 715.4 and 728.0 eV^52^. Furthermore, the two small satellite peaks revealed that the surface of the magnetic nanocomposites was slightly oxidized in the environment^53^.

### Electrochemical characterization and SC performance

**Fig. 7:**
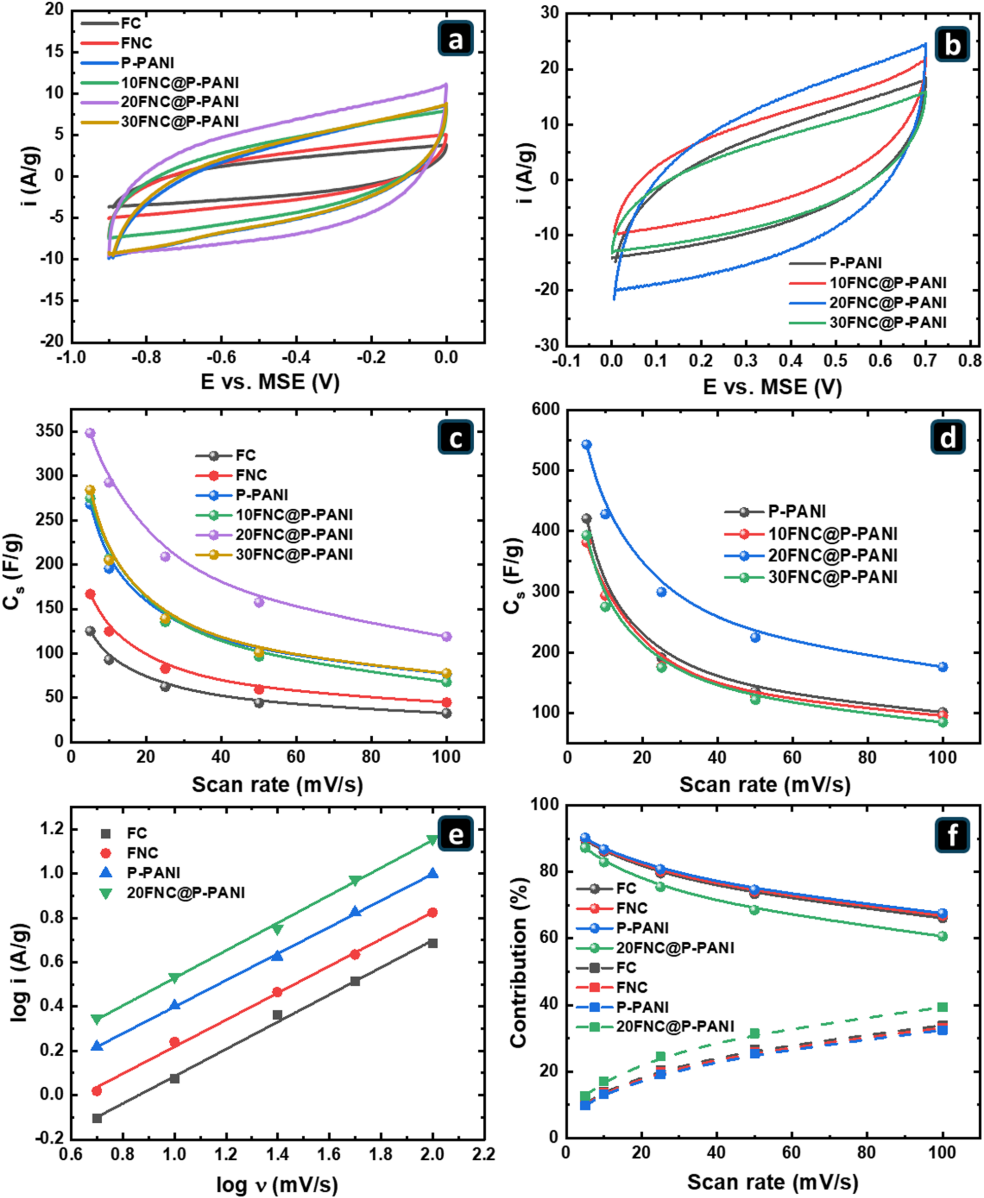
CV curves at 50 mV/s of the FC, GNC, P-PANI, and 20FNS@P-PANI electrodes within potential ranges of (**a**) -0.9 to 0.0 V and (**b**) 0.0 to 0.7 versus MSE in 1 M Li_2_SO_4_ at RT. Specific capacitance (C_s_) of prepared electrodes calculated from the CVs with a potential range of (**c**) -0.9 to 0.0 V and (**d**) 0.0 to 0.7 V versus MSE at different scan rates. (**e**) Power-Law plot for the electrodes (cathodic performance). (**f**) the contribution (%) of non-diffusion-controlled (dashed line + Square symbol) and diffusion-controlled (solid line + Spherical symbol) charge storage.

To assess the supercapacitive (SC) performance of the as-synthesized samples, cyclic voltammetry (CV), galvanostatic charge/discharge (GCD), and electrochemical impedance spectroscopy (EIS) measurements were performed. Figures S3 and S4 show the CVs of the fabricated composites as cathode and anode electrodes at a potential range from − 0.9 to 0.0 V versus MSE and 0.7 to 0.0 V versus MSE at different scan rates from 5 to 100 mV/s in 1.0 M Li_2_SO_4_ as electrolyte. For comparison, Fig. 7a and b display the CVs of the cathode and anode electrodes at a scan rate of 50 mV/s.

The CV curves for all samples showed rectangular shapes, characteristic of a dominant EDLC behavior, attributed to the intercalation of Li^+^ ions within the fabricated materials^54^. However, minor peaks were visible in some samples only at lower scan rates (Figure S5), suggesting very minor contributions from surface redox (pseudocapacitive) reactions associated with Fe-containing species or nitrogen functionalities introduced during pyrolysis. The FC electrode exhibited a minimal current response compared to the FNC and P-PANI due to the presence of nitrogen heteroatoms within the carbon structure (N-doping) that formed during the pyrolysis of the frameworks containing amino groups (NH_2_-MIL101(Fe)) and PANI^55^. Nitrogen doping is a highly effective strategy to enhance the electrochemical performance of carbon materials by increasing electronic conductivity, introducing additional ion storage sites, and improving capacitance^56^. Due to its higher electronegativity (3.04) compared to carbon (2.55) and its extra valence electrons. Furthermore, nitrogen atoms distort the carbon lattice when incorporated, creating local strain in the carbon rings. This distortion, along with the delocalization of nitrogen’s lone electron pair, improves electron transport and surface reactivity^57,58^. Moreover, the additional free electrons from nitrogen doping further boost the conductivity of the carbon frameworks^59^. Composing FNC and P-PANI further enhanced their performance. The presence of P-PANI in the fabricated composite not only improves conductivity but also provides a high surface area for FNC dispersion. Among the FNC@P-PANI composites, 20FNC@P-PANI showed a CV loop with the most significant integral area, indicating the highest capacitance and exhibiting a maximum synergistic effect between P-PANI and FNC. It is worth noting that increasing the FNC content in the composite to 30 wt% has decreased the specific capacitance. The high FNC content might agglomerate and block the porous structure of P-PANI, thereby masking its effect on enhancing pseudocapacitance.

Figure 7c and d illustrate the dependence of the calculated mass-specific capacitance (Cs) on scan rate for the fabricated electrodes as cathodes and anodes, respectively. The 20FNC@P-PANI electrode showed the highest Cs as both cathode and anode, showing 348.41 F/g in the potential range of -0.9 to 0 V and 543.3 F/g in the potential range of 0 to 0.7 at a scan rate of 5 mV/s. The Cs of the 20FNC@P-PANI cathode and anode dropped to 118.98 F/g and 200 F/g, respectively, when the scan rate was increased to 100 mV/s. This is a common phenomenon because ions in the electrolyte do not have enough time to penetrate the complex micropores of the electrodes (diffusion-limited) at high scan rates. The power law was used to further understand the contributions of the surface (non-diffusion-limited) and the bulk (diffusion-limited) to the energy storage capacity of the prepared electrodes, as illustrated in Eq. 5^60^.

5 $$\:\mathrm{log}\mathrm{I}\:\left(\mathrm{V}\right)=\mathrm{a}+\mathrm{b}\mathrm{log}{\upnu\:}$$

Where I (V) is the voltage-dependent current value at a given scan rate (ν), and a and b are two adjustable parameters.

Figure 7e shows a plot of log I versus log v, using current values at -0.4 V at different scan rates to illustrate cathodic behavior. In the power law, when b equals 1, that indicates a surface-controlled process; when b equals 0.5, it indicates a diffusion-limited bulk process^60^. According to Fig. 7e, the calculated b values were 0.613, 0.609, 0.599, and 0.622 for FC, FNC, P-PANI, and 20FNC@P-PANI, respectively, indicating that a combination of surface and diffusion-bulk processes controls the charge storage mechanism. However, the diffusion-limited behavior is more dominant because the b value is close to 0.5. To determine quantitatively the contribution of each mechanism (diffusion-limited (bulk)) versus non-diffusion-limited (surface)) to the capacitive behavior of the fabricated electrodes, Eqs. 6 and 7 were applied^61^.

6 $$\:\mathrm{I}\left(\mathrm{V}\right)={\mathrm{k}}_{1}{\upnu\:}+{\mathrm{k}}_{2}{{\upnu\:}}^{0.5}$$

7 $$\:\mathrm{I}\left(\mathrm{V}\right)/{{\upnu\:}}^{0.5}={\mathrm{k}}_{1}{{\upnu\:}}^{0.5}+{\mathrm{k}}_{2}$$

where; $\:{\mathrm{k}}_{1}{\upnu\:}$ and $\:{\mathrm{k}}_{2}{{\upnu\:}}^{0.5}$ represent the current contribution of the surface and bulk, respectively.

The values of k_1_ and k_2_ are calculated by profiling the $\:\mathrm{I}\left(\mathrm{V}\right)/{{\upnu\:}}^{0.5}\:$versus $\:{{\upnu\:}}^{0.5}$ curve. Using k_1_ and k_2_ values for each electrode, the charge storage contribution of each mechanism is represented in Fig. 7f, with the non-diffusion-controlled (surface) represented as a dashed line + Square symbol and the diffusion-controlled (bulk) contribution represented by a solid line + Spherical symbol. Figure 7f indicates that the charge storage is mainly attributed to the diffusion (bulk) process, agreeing with the b values obtained from Fig. 7e. For instance, at a scan rate of 5 mV/s, the 20FNC@P-PANI electrode showed a non-diffusion-limited (surface) contribution of 12.7% to the energy storage of the material, compared to 87.3% related to the diffusion-limited (bulk) contribution. For FC, FNC, and P-NC electrodes, the surface contributions are 10.3%, 9.9%, and 9.7%, respectively, while the remaining 89.7%, 90.1%, and 90.3% are contributions from the bulk, respectively. Slow scan rates allow ions to diffuse to deeper regions within the electrodes, making not only easy-to-access sites available but also diffusion-limited sites in the bulk. As the scan rate increases, the overall capacitance decreases. In contrast, the bulk contribution decreases because ions have less time to diffuse through it, while the surface contribution increases. Furthermore, the 20FNC@P-PANI electrode has the highest non-diffusion-limited (surface) contribution at all scan rates, confirming the enhancing effect of the FNC and P-PANI on the overall capacitive behavior by inhibiting stacking, providing mixed oxidation states, and functionalization, which in turn maximizes the surface-to-bulk ratio, increases hydrophilicity, and results in a high amount of oxygen vacancies^62^.

**Fig. 8:**
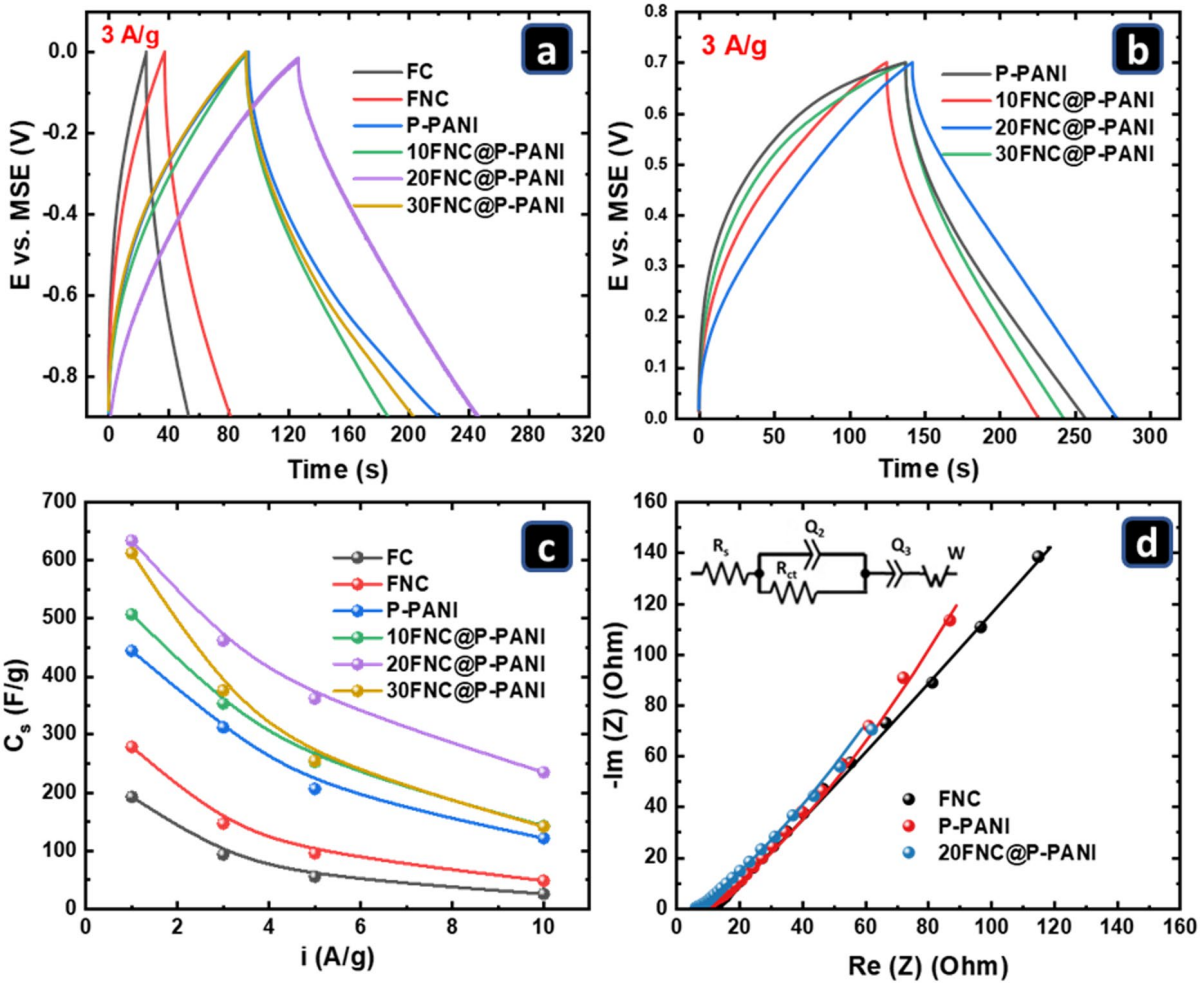
GCD at the potential range of (**a**) -0.8–0 and (**b**) 0–0.7 at 3 A/g, employing a three-electrode system, (**c**) Specific capacitance at several current densities, (**d**) Nyquist plot of the EIS measurements at OCP, of FNC, P-PANI, and 20FNC@P-PANI electrodes in 1 M Li_2_SO_4_ at RT.

Galvanostatic charge/discharge (GCD) measurements are essential for evaluating the performance of the prepared materials in supercapacitor applications. Figures S6 and S7 show the GCD responses of the prepared electrodes in the cathodic (-0.9 to 0.0 V versus MSE) and anodic (0.7 to 0.0 V versus MSE) potential ranges at specific current densities of 1–10 A/g. for comparison, Fig. 8a and b show the GCD graphs for the fabricated composites in the cathodic and anodic potential ranges at a current density of 3 A/g. All GCD plots are quasi-triangular, indicating fast and capable charge transfer and high electrical conductivity^63^.

Figure 8c shows the mass-specific capacitance (Cs) as a function of applied current density calculated from Figure S6 (cathodic) as a representative for both the cathodic and anodic general behavior. The 20FNC@P-PANI electrode attained the highest Cs (634 F/g), about 3.3 times that of FC (193 F/g), 2.3 times that of FNC (279 F/g), and 1.4 times that of P-PANI (444 F/g), at 1 A/g. This can be related to the presence of nitrogen atoms derived from the pyrolysis of amine frameworks (NH_2_-MIL-101(Fe)) and PANI, which improve electron conductivity, thereby influencing the electron distribution and polarization of some bonds^55,64^. Moreover, nitrogen doping can shift the carbon’s Fermi level into the valence band, making it easier for electrons to transfer and creating surface dipoles on the FNC@P-PANI composite that attract charged particles, facilitating the easier propagation of Li^+^ ions into the 20FNC@P-PANI. Note that the specific capacitance of all samples decreases with increasing current density. This drop is due to the limitation of Li^+^ ion diffusion, which could be overcome by increasing the Li^+^ concentration. However, 20FNC@P-PANI retained a high Cs even at a very high applied current, compared with previously reported values (Table 1)^65–71^.

**Table 1:** A comparison of the specific capacitance results from this work and those from earlier studies on 20FNC@P-PANI nanocomposites.

Materials	Specific capacitance (F/g) at 1 A/g	Electrolyte	References
MIL-101(Fe)	7.56	3.0 M KOH	^65^
MIL-101(Fe)-C (600 °C)	48.3	3.0 M KOH	^65^
PANI (850 °C)	419.8 at 0.2 A/g 343.2 179.8	1.0 M H_2_SO_4_ 6.0 M KOH 0.5 M K_2_SO_4_	^66^
HPDSC (800 °C)	245.0	0.5 M Na_2_SO_4_	^67^
PNCNFs (850 °C)	198.0	6.0 M HOH	^68^
Fe_2_O_3_/MnO_2_	216.0	1.0 M KOH	^69^
MoS_2_/Fe_2_O_3_/G	98.2	2.0 M KOH	^70^
Fe_2_O_3_-ACC	295.56	3.0 M KOH	^71^
**20FNC@P-PANI**	**633.96 (Anode)** **693.6 (Cathode)**	**1.0 M Li**_**2**_ **SO**_**4**_	**This work**

To further understand the performance of the synthesized materials, the number of electrochemically active sites was calculated based on the GCD performance at a current density of 1 A/g using Eq. 8, shown in Figure S8, where Q is the charge density (C/g), n is the number of electrons, and F is Faraday’s constant (96,485 C/mol)^72^.

8 $$\:\mathrm{N}\mathrm{u}\mathrm{m}\mathrm{b}\mathrm{e}\mathrm{r}\:\mathrm{o}\mathrm{f}\:\mathrm{A}\mathrm{c}\mathrm{t}\mathrm{i}\mathrm{v}\mathrm{e}\:\mathrm{s}\mathrm{i}\mathrm{t}\mathrm{e}\mathrm{s}=\frac{\mathrm{Q}}{\mathrm{n}\mathrm{F}}$$

The numbers of active sites are 1.5, 2.5, 4.5, 4.7, 5.9, and 5.5 mmol/g for FC, FNC, P-PANI, 10FNC@P-PANI, 20FNC@P-PANI, and 30FNC@P-PANI, respectively. This confirms the superior performance of the 20FNC@P-PANI electrode, which shows the highest number of active sites.

Figure 8d shows the Nyquist plots of FNC, P-PANI, and 20FNC@P-PANI. The circuit includes R_1_ (solution and contact resistance), R_2_ (charge transfer resistance), Q_2_ & Q_3_ (constant phase elements related to surface and bulk capacitive behavior), and W (diffusion limitations)^62^. The slope of the EIS response at low frequencies could indicate the degree of diffusion limitations. It is known as Warburg impedance, which originates from the frequency-dependent diffusion of ions to the electrode-electrolyte interface. A slope of 1 indicates a fully diffusion-controlled process, while higher slopes indicate less diffusion limitation. In the case of FNC, P-PANI, and 20FNC@P-PANI electrodes, the slope was found to be 0.79, 0.85, and 0.77, respectively, indicating the domination of the diffusion-limitation mechanism.

**Fig. 9:**
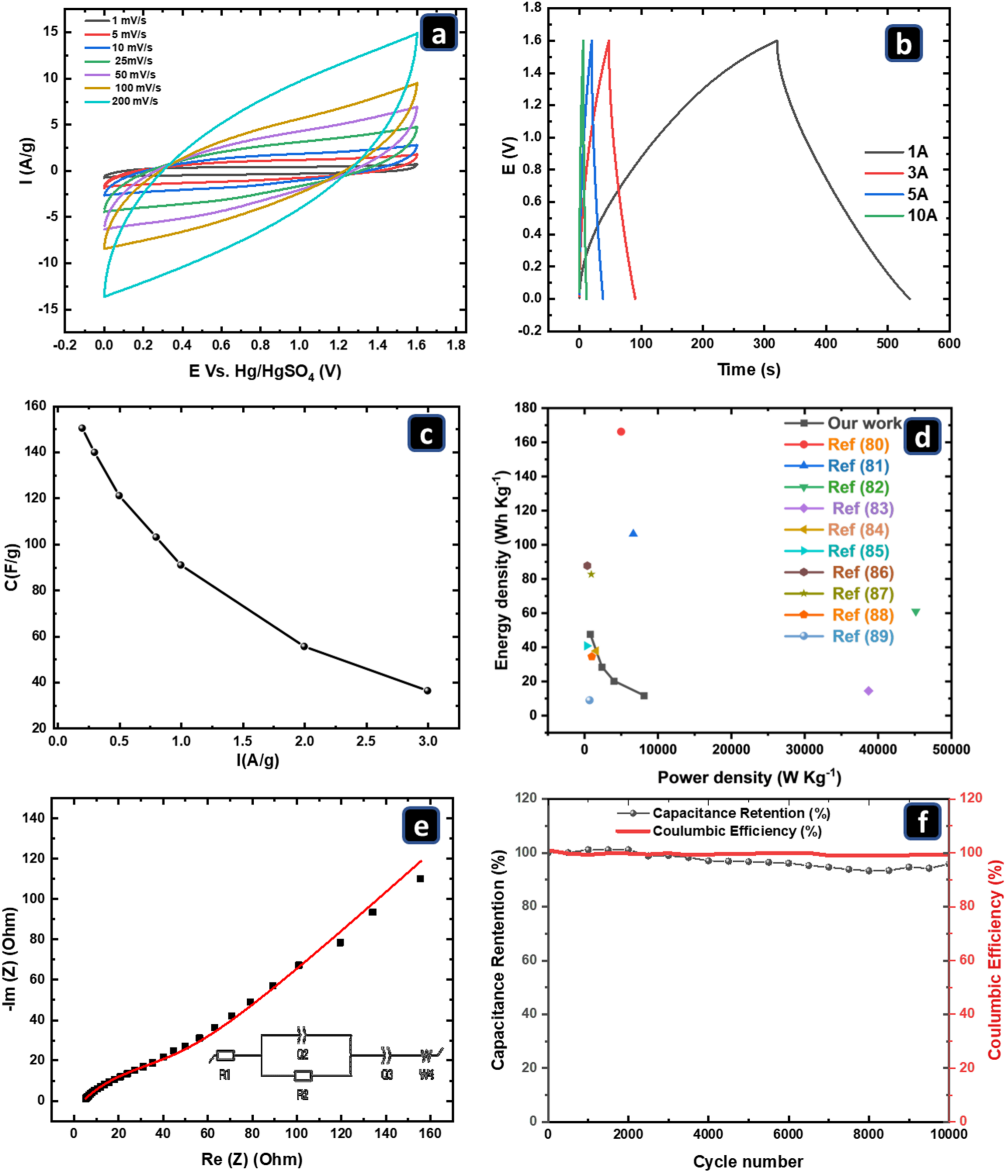
(**a**) CV curves at different scan rates, (**b**) GCD plots at different current densities, (**c**) specific capacitance (Cs) and (**d**) Ragone plot at different current densities and compared earlier studies at a current density of 1 A/g, (**e**) Nyquist plot of the EIS results at OCP, and (**f**) The stability after 10,000 GCD cycles at a current density of 10 A/g for the 20FNC@P-PANI symmetric device at RT and using 1.0 M Li_2_SO_4_ in as an electrolyte.

The CV and GCD results of the 20FNC@P-PANI electrode in the 3-electrode setup, shown previously, demonstrate that it performs best and indicate its suitability for use as both the positive and negative electrodes. Therefore, its electrochemical performance was evaluated using a symmetric two-electrode system (20FNC@P-PANI//20FNC@P-PANI) with 1.0 M Li_2_SO_4_ as the electrolyte. Figure 9a shows the CV curves of the symmetric 20FNC@P-PANI supercapacitor device at various scan rates (1-200 mV/s) under the voltage window from 0 to 1.6 V. All voltammograms of the assembled device illustrated ideal rectangular shapes without noticeable reversible redox peaks^73^.

The GCD curves of the 20FNC@P-PANI//20FNC@P-PANI device at current densities of 1, 3, 5, and 10 A/g are illustrated in Fig. 9b. The calculated specific capacitances at these current densities of 133, 79, 56, and 33 F/g, respectively, are shown in Fig. 9c. All GCD plots showed almost symmetrical behavior without a discernible IR drop, indicating outstanding coulombic efficiency, capacitive behavior, and good electrochemical reversibility^74^. The nitrogen heteroatom in P-PANI and FNC nanoparticles enhances charge density, resulting in excellent charge/discharge reversibility for the 20FNC@P-PANI materials due to improved electrical conductivity. Furthermore, porous surfaces and an interconnected network of FNC within the pyrolyzed PANI were observed, suggesting good interaction between the FNC and the P-PANI.

The energy and power densities are critical performance metrics for supercapacitors and can be calculated from GCD plots using Eqs. 3 and 4, and presented in Ragone’s plot (Fig. 9d). The assembled symmetric 20FNC@P-PANI supercapacitor delivers an energy density of up to 48 Wh/kg and a power density of 790 W/kg at a current density of 1 A/g. The fabricated device shows energy and power densities of 11.6 Wh/Kg and 8116 W/Kg at a high current density of 10 A/g, implying fast ion and electron propagation. It is worth mentioning that the achieved energy density for the 20FNC@P-PANI electrode is significantly higher than those reported in the literature, which is a promising result for supercapacitor applications. For instance, Fe_x_O_y_/C with Cr_2_O_3_/C composites derived from frameworks exhibited an energy density of 9.6 Wh/kg at a power density of 800 W/kg^75^. The synthesized FeX@PC-3 exhibited a maximum energy density of 10.5 Wh/kg at a power density of 250 W/kg^76^. The asymmetric system based on Fe_3_O_4_ and reduced GO showed an energy density of 18.3 Wh/kg at 351 W/kg^77^. Moreover, the PANI/MIL-101-based supercapacitor exhibited an energy density of 7 Wh/kg at a power density of 2000 W/kg^78^. A comparison of the assembled cell with previously reported results from earlier studies at a current density of 1 A/g is shown in a Ragone plot (Fig. 9d)^79–88^. Furthermore, Table S2 compares the power and energy density results from this work with those from earlier studies.

The enhanced performance of the symmetric 20FNC@P-PANI supercapacitor device may be attributed to the synergistic stacking inhibition, reduction, and functionalization of the synthesized composite with 2-aminoterephthalic acid, in addition to its wider 1.6 V working potential window.

The electrochemical impedance spectrum of the synthesized 20FNC@P-PANI was measured in the frequency range of 0.1–10^6^ Hz at open circuit potential with an amplitude of 5 mV. The Nyquist plots in Fig. 9e show that the measured Rs was approximately 5.3 Ω, attributed to the series solution resistance and the active material/current collector contact resistance. The presence of a very small semicircle at high frequencies, with the Warburg behavior dominating the EIS response, indicates a significant diffusion-limited process in the energy storage mechanism of 20FNC@P-PANI materials, confirming the three-electrode measurements presented in Fig. 7f and e.

The cycle life test of the 20FNC@P-PANI electrodes was done by performing GCD at a current density of 10 A/g for 10,000 cycles, as shown in Fig. 9f. The specific capacitance of the two-electrode system is maintained at 100.1% after 1000 cycles, attributed to the activation process of the electrode, and decreases slightly to 95.86% after 10,000 cycles, suggesting the excellent cycle stability of the assembled device^89^. The calculated Coulombic efficiency is maintained at 100%, indicating the excellent cycling stability of the 20FNC@P-PANI electrode compared to those reported in the literature^62^. The high capacitance and stability of the fabricated device based on 20FNC@P-PANI electrode highlight how promising these electrode materials are for electrochemical supercapacitance applications.

## Conclusion

In this work, the FNC@P-PANI composites were successfully synthesized, demonstrating enhanced interfacial binding between the FNC and P-PANI nanostructures. Among them, the 20FNC@P-PANI composite exhibited exceptional supercapacitor performance, achieving a high specific capacitance of 634 F/g at a current density of 1 A/g. This value surpassed those of pure P-PANI (506 F/g) and FNC (147 F/g) at 3 A/g, highlighting the synergistic effect of combining pyrolyzed frameworks with conductive P-PANI. The symmetric supercapacitor device assembled using the 20FNC@P-PANI electrode exhibited a remarkable specific capacitance of 134 F/g and demonstrated excellent cycling stability, retaining 95.86% of its initial capacitance after 10,000 charge-discharge cycles. Additionally, the device showed impressive energy and power densities of 48 Wh/kg and 790 W/kg, respectively, at a current density of 1 A/g. These outstanding properties stem from the uniform distribution of nitrogen heteroatoms across the nanocomposite surface and the synergistic interactions between Fe_3_O_4_ and the P-PANI matrix, as indicated by the FT-IR, EDS, and XPS results. Overall, the 20FNC@P-PANI composite shows promising behavior as a high-performance electrode material for practical energy storage applications, owing to its excellent conductivity, stability, and capacitive behavior.

## Supplementary Information

Below is the link to the electronic supplementary material.

Supplementary Material 1

## Data Availability

The datasets that were generated and subsequently analyzed during the current study are presented in their entirety within this published article. All necessary supporting data, including figures, tables, and primary findings, are included in the main text and its accompanying supplementary information files, where applicable.
