# Supplementary material for: Iron oxide decorated nitrogen doped carbon derived from iron MOFs and polyaniline as binder free electrode for symmetric supercapacitors
Source: Sci Rep. 2026 Mar 9;16:8615. doi: 10.1038/s41598-026-39173-4 (PMC12976114; doi:10.1038/s41598-026-39173-4)
Supplement: Supplementary file 1 — Supplementary Material 1 [file 41598_2026_39173_MOESM1_ESM.docx]

**Iron Oxide Decorated Nitrogen Doped Carbon Derived from Iron MOFs and Polyaniline as Binder Free Electrode for Symmetric Supercapacitors**

Aya A. El-Ashry^a, b^, Dalia M. El-Gendy^c, b^, Mina Shawky Adly^a^, Ehab N. El Sawy^b*^, and Sohier A. El-Hakam^a^

^a^ Chemistry Department, Faculty of Science, Mansoura University, Egypt, 35516

^b^ Department of Chemistry, School of Science and Engineering, The American University in Cairo, Egypt, 11835

^c^ Physical Chemistry Department, National Research Centre, Dokki, Giza, Egypt

*Corresponding Author: [ehab.elsawy@aucegypt.edu](mailto:ehab.elsawy@aucegypt.edu)

**Tables:**

**Table S1:** Chemical composition of 20FNC@P-PANI composite, as determined by EDS and XPS analysis, in at%.

| **Element** | **EDS (At%)** | **XPS (At%)** |
| --- | --- | --- |
| **C** | **84.9** | **81.39** |
| **N** | **10.21** | **11.1** |
| **O** | **4.94** | **7.17** |
| **Fe** | **0.45** | **0.34** |

**Figures:**

**
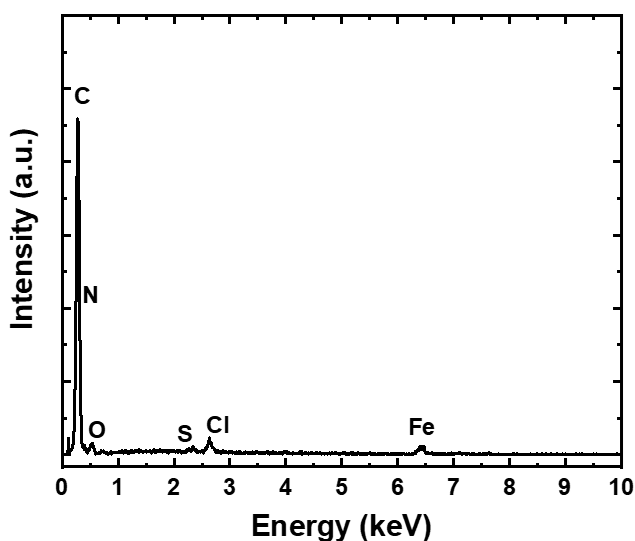
**

**Figure S1.** The EDX spectrum of the 20FNC@P-PANI composites.

**
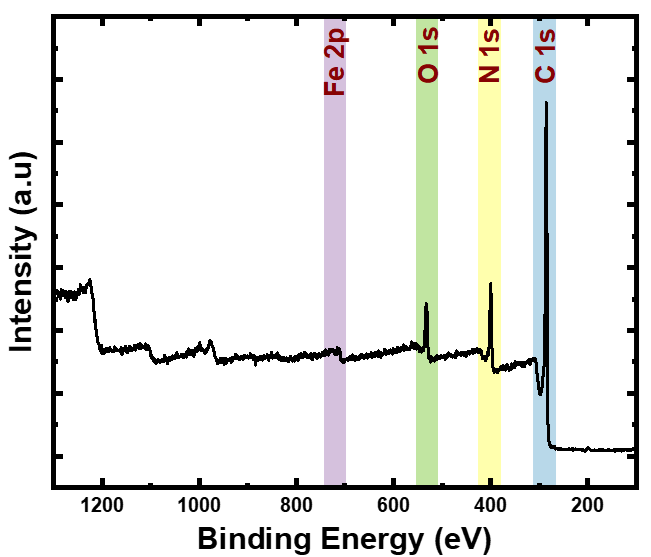
**

**Figure S2.** Full survey XPS spectrum of 20FNC@P-PANI

**
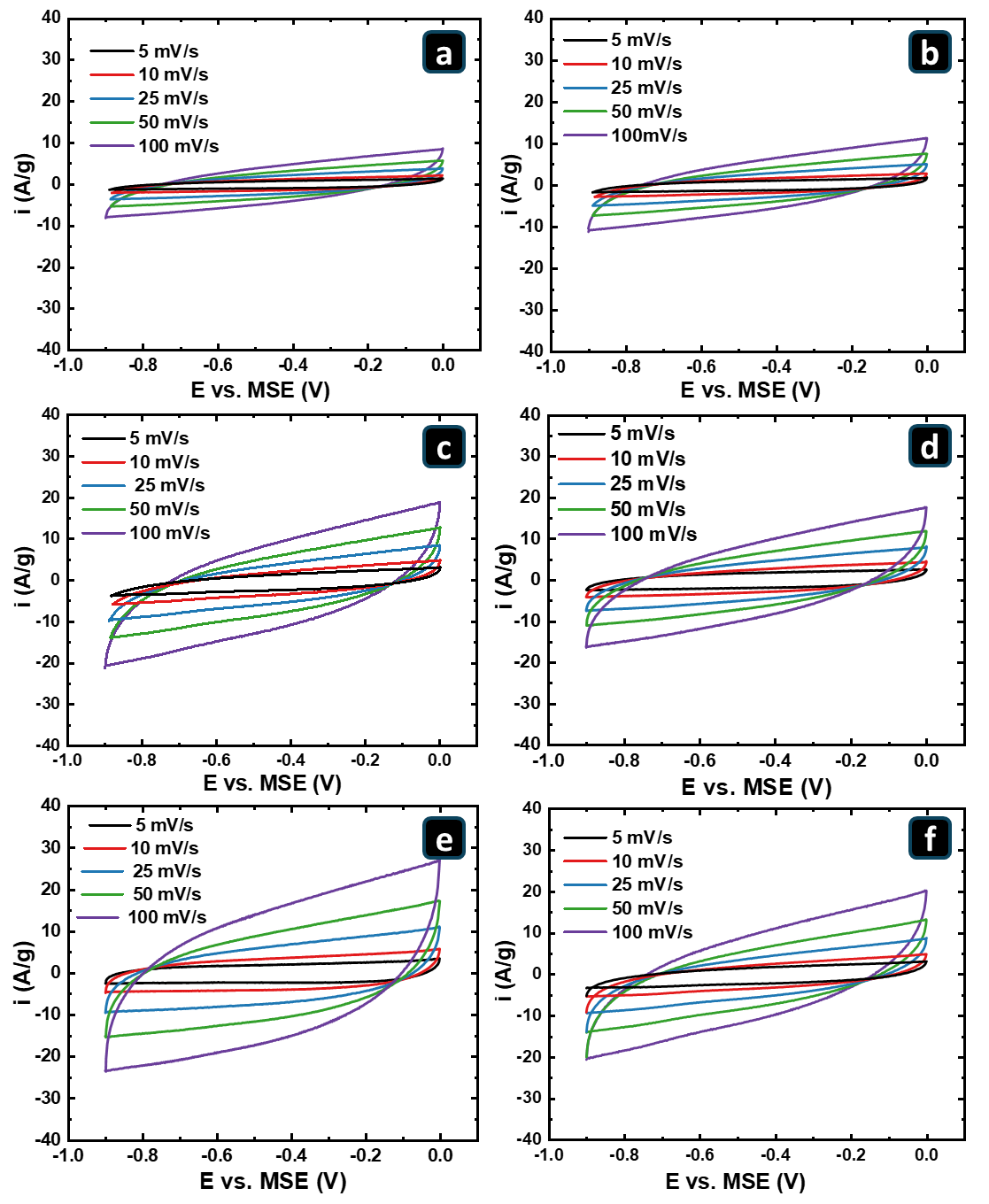
Figure S3.** CVs of (a) FC, (b) FNC, (c) P-PANI, (d) 10FNC@P-PANI, (e) 20FNC@P-PANI, and (f) 30FNC@P-PANI in 1.0 M Li_2_SO_4_, using different scan rates from 5 to 100 mV/s at a potential range of $-$0.9 to 0.0 V vs. MSE


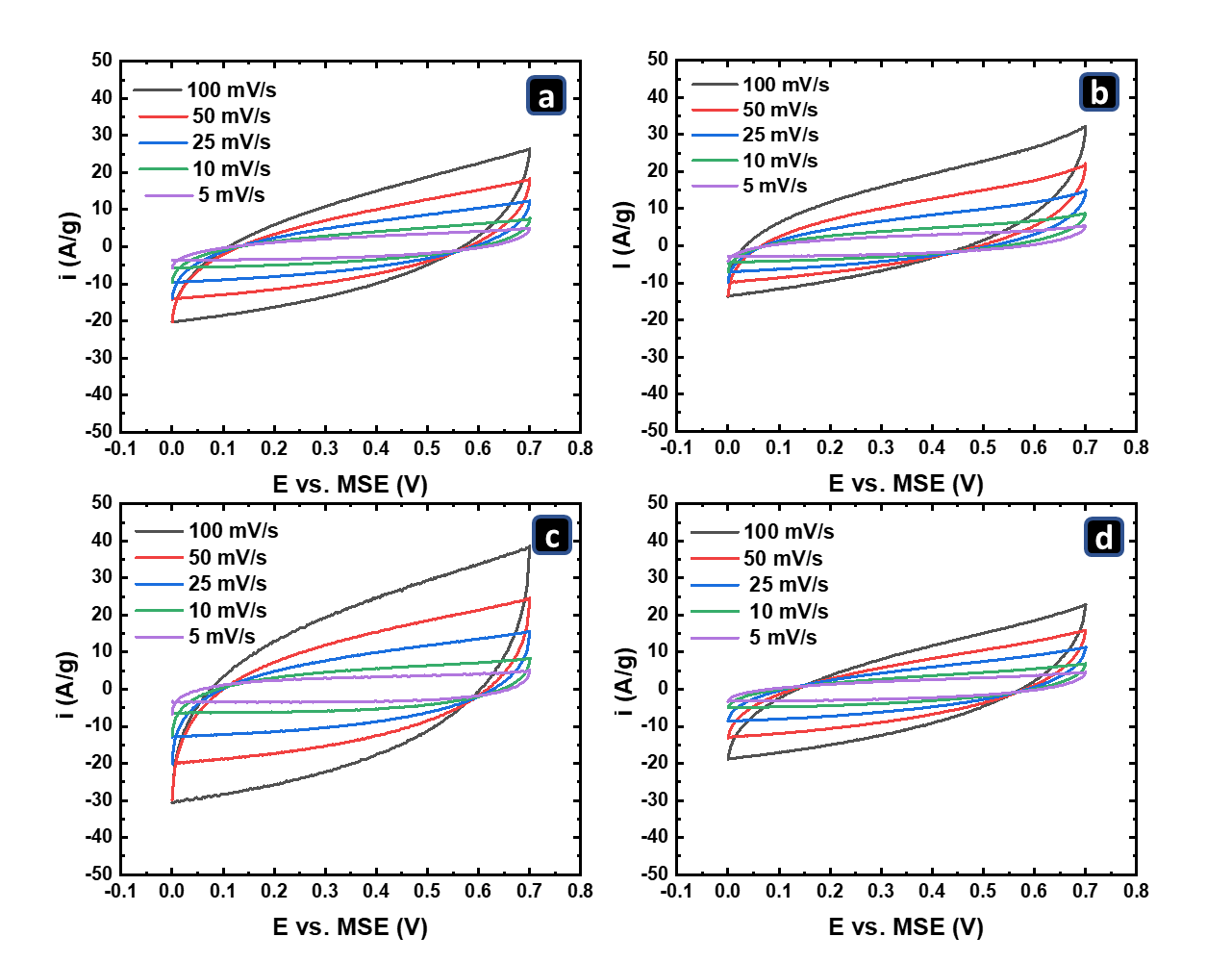


**Figure S4.** CVs of (a) P-PANI, (b) 10FNC@P-PANI, (c) 20FNC@P-PANI, and (d) 30FNC@P-PANI in 1.0 M Li_2_SO_4_, using different scan rates from 5 to 100 mV/s at potential range from 0.0 to 0.7 V vs. MSE


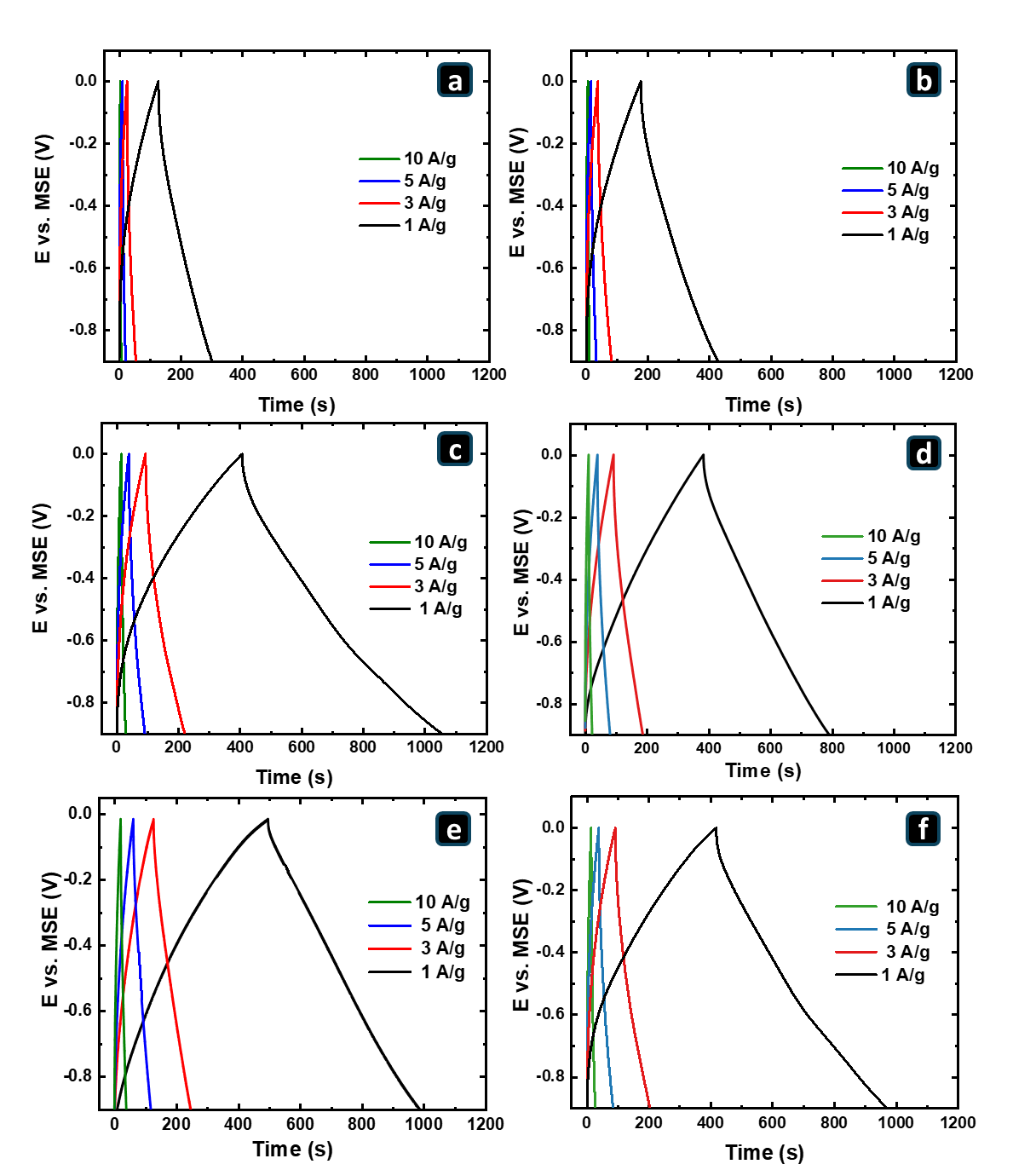


**Figure S5.** The GCD responses of (a) FC, (b) FNC, (c) P-PANI, (d) 10FNC@P-PANI, (e) 20FNC@P-PANI, and (f) 30FNC@P-PANI in 1.0 M Li_2_SO_4_, using different current densities from 1 to 10 A/g at a potential range of -0.9 to 0.0 V vs. MSE


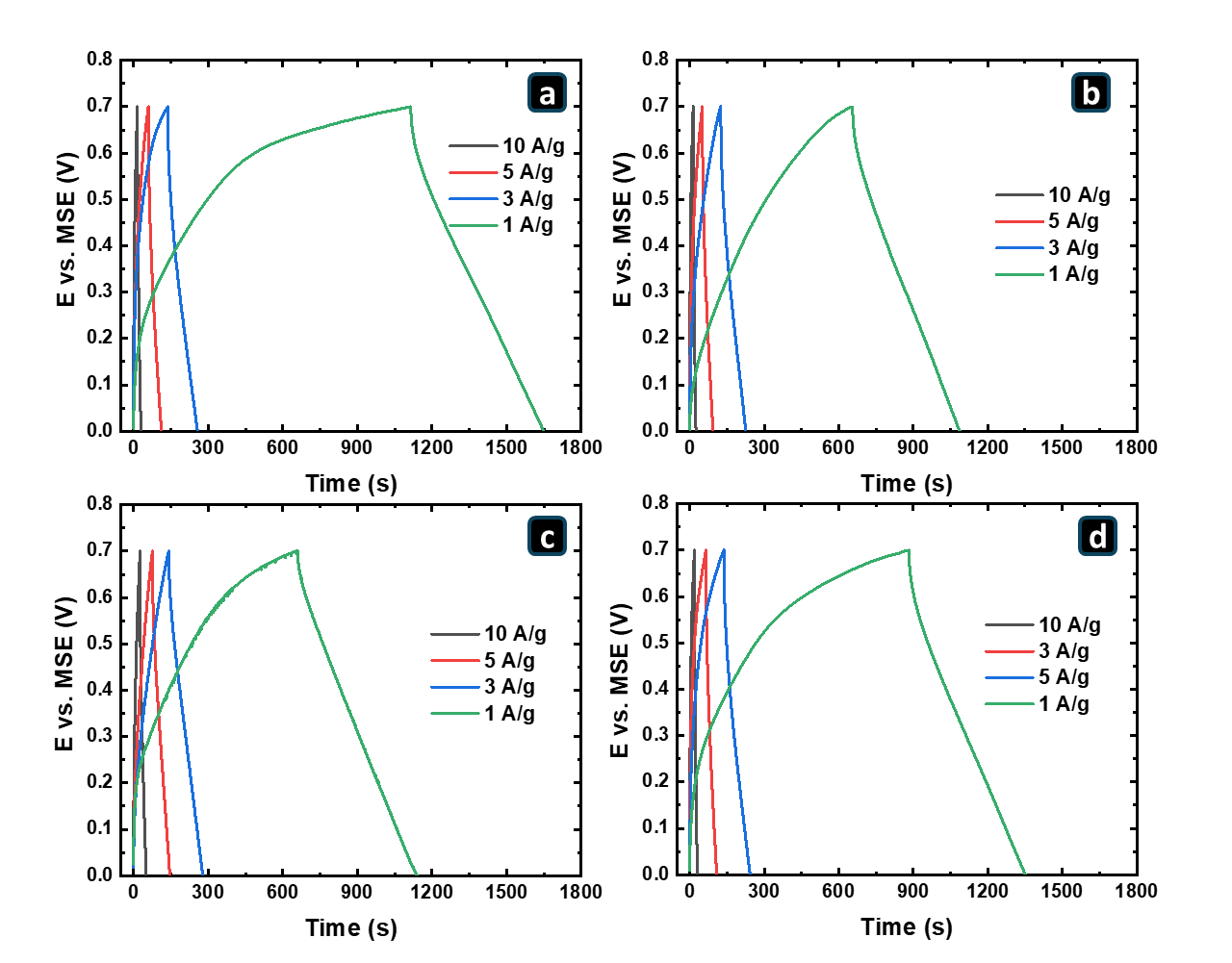


**Figure S6.** The GCD responses of (a) P-PANI, (b) 10FNC@P-PANI, (c) 20FNC@P-PANI, and (d) 30FNC@P-PANI in 1.0 M Li_2_SO_4_, using different current densities from 1 to 10 A/g at a potential range of 0.0 to 0.7 V vs. vs. MSE

| **Electrode material** | **Energy Density (Wh/kg)** | **Power Density (kW/kg)** | **Configuration/Notes** | **References** |
| --- | --- | --- | --- | --- |
| PANI/Fe_3_O_4_ (waste toner derived) | 166.2 | 5 | Symmetric, 1.0 M KOH, 2-electrode | [1] |
| Fe_3_O_4_@Fc-GO/PANI | 106.2 | 6.66 | Symmetric, 2-electrode, 1.0 M H_2_SO_4_ | [2] |
| rGO-CNT-Fe_3_O_4_-PANI | 60.8 | 45.2 | Symmetric, flexible, 2-electrode, 1.0 M H_2_SO_4_ | [3] |
| MnO_2_/Fe_3_O_4_ (1 : 1) | 14.17 | 38.75 | Asymmetric, 1m KOH | [4] |
| Fe_2_O_3_/N-PC | 37.6 | 1.6 | Symmetric, 6.0 M KOH | [5] |
| NG/Cdots/Fe₃O₄ (film) | 40.6 | 0.320 | Symmetric, 1.0 M NaCl | [6] |
| Graphene/Fe_3_O_4_ | 87.6 | 0.394 | Asymmetric, 1.0 M Na_2_SO_4_ | [7] |
| Fe_3_O_4_/rGO | 82.5 | 0.930 | Asymmetric, 1.0 M KOH | [8] |
| Hierarchical dumbbell-like Fe_3_O_4_/C powder | 34.3 | 1004 | Symmetric, 1.0 M KOH | [9] |
| FSS-SCs | 8.7 | 0.694 | Symmetric, 1.0 M Na_2_SO_4_ | [10] |
| 20FNC/P-PANI | 48 | 0.790 | Symmetric, 1.0 M Li_2_SO_4_ | This work |

Table S1. A comparison of the power and energy density results from this work and those from earlier studies.

**Figure S7.** Number of active sites of different prepared electrodes at 1 A/g

.


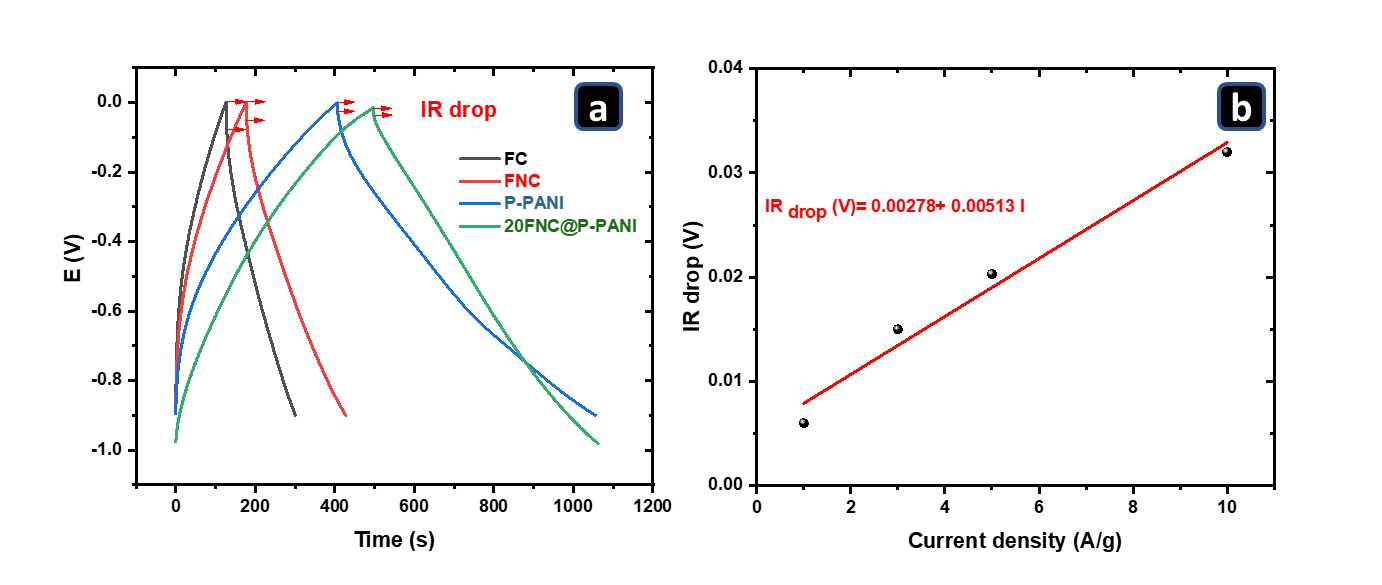


**Figure S8**. (a) The curve for the IR drop at a current density of 1 A/g and (b) IR drop for 20FNC@P-PANI at different current densities 1, 3, 5, and 10 A/g.

**References**

1. Sharma, D. and S. Kumar, *Waste toner derived Fe_3_O_4_ nanoparticles embedment into PANI matrix as an advanced electrode for supercapacitor.* Physica Scripta, 2025. **100**(5).

2. Payami, E. and R. Teimuri-Mofrad, *A novel ternary Fe_3_O_4_@Fc-GO/PANI nanocomposite for outstanding supercapacitor performance.* Electrochimica Acta, 2021. **383**.

3. Ao, J., R. Miao, and J. Li, *Flexible solid-state supercapacitor based on reduced graphene oxide-enhanced electrode materials.* Journal of Alloys and Compounds, 2019. **802**: p. 355-363.

4. Dessie, Y., et al., *Electrochemical performances of MnO_2_/Fe_3_O_4_/activated carbon ternary composites for supercapacitor and direct ethanol fuel cell catalyst application.* RSC Adv, 2025. **15**(21): p. 16493-16509.

5. Yang, Z., et al., *Fe_2_O_3_ Embedded in N-Doped Porous Carbon Derived from Hemin Loaded on Active Carbon for Supercapacitors.* Molecules, 2023. **29**(1).

6. Fite, M.C., P.-J. Wang, and T. Imae, *Symmetric and Asymmetric Supercapacitors of ITO Glass and Film Electrodes Consisting of Carbon Dot and Magnetite.* Batteries, 2023. **9**(3).

7. Sheng, S., et al., *Fe_3_O_4_ nanospheres in situ decorated graphene as high-performance anode for asymmetric supercapacitor with impressive energy density.* J Colloid Interface Sci, 2019. **536**: p. 235-244.

8. Lin, T.W., C.S. Dai, and K.C. Hung, *High energy density asymmetric supercapacitor based on NiOOH/Ni_3_S_2_/3D graphene and Fe_3_O_4_/graphene composite electrodes.* Sci Rep, 2014. **4**: p. 7274.

9. Lan, J., et al., *Hierarchical dumbbell-like Fe_3_O_4_/C electrode for the supercapacitor.* Materials Letters, 2023. **331**.

10. Gund, G.S., et al., *Low-cost flexible supercapacitors with high-energy density based on nanostructured MnO_2_ and Fe_2_O_3_ thin films directly fabricated onto stainless steel.* Sci Rep, 2015. **5**: p. 12454.
